# Anti-neoplastic drugs increase caveolin-1-dependent migration, invasion and metastasis of cancer cells

**DOI:** 10.18632/oncotarget.22955

**Published:** 2017-12-05

**Authors:** Natalia I. Díaz-Valdivia, Claudia C. Calderón, Jorge E. Díaz, Lorena Lobos-González, Hugo Sepulveda, Rina J. Ortíz, Samuel Martinez, Veronica Silva, Horacio J. Maldonado, Patricio Silva, Sergio Wehinger, Verónica A. Burzio, Vicente A. Torres, Martín Montecino, Lisette Leyton, Andrew F.G. Quest

**Affiliations:** ^1^ Cellular Communication Laboratory, Center for Molecular Studies of the Cell (CEMC), Advanced Center for Chronic Diseases (ACCDiS), Faculty of Medicine, Universidad de Chile, Santiago, Chile; ^2^ Institute for Research in Dental Sciences, Faculty of Dentistry, Universidad de Chile, Santiago, Chile; ^3^ Fundación Ciencia & Vida, Santiago, Chile; ^4^ Faculty of Health Sciences, University of Talca, Interdisciplinary Excellence Research Program Healthy Ageing (PIEI-ES), Talca, Chile; ^5^ Gene Regulation Laboratory, Center for Biomedical Research, Faculty of Biological Sciences and Faculty of Medicine, Universidad Andrés Bello, Santiago, Chile; ^6^ Faculty of Biological Sciences, Universidad Andrés Bello, Santiago, Chile; ^7^ Universidad Bernardo O Higgins, Facultad de Salud, Departamento de Ciencias Químicas y Biológicas, Santiago, Chile

**Keywords:** caveolin-1, epigenetic silencing, chemotherapy, cell signaling, reactive oxygen species

## Abstract

Expression of the scaffolding protein Caveolin-1 (CAV1) enhances migration and invasion of metastatic cancer cells. Yet, CAV1 also functions as a tumor suppressor in early stages of cancer, where expression is suppressed by epigenetic mechanisms. Thus, we sought to identify stimuli/mechanisms that revert epigenetic CAV1 silencing in cancer cells and evaluate how this affects their metastatic potential. We reasoned that restricted tissue availability of anti-neoplastic drugs during chemotherapy might expose cancer cells to sub-therapeutic concentrations, which activate signaling pathways and the expression of CAV1 to favor the acquisition of more aggressive traits. Here, we used *in vitro* [2D, invasion] and *in vivo* (metastasis) assays, as well as genetic and biochemical approaches to address this question. Colon and breast cancer cells were identified where CAV1 levels were low due to epigenetic suppression and could be reverted by treatment with the methyltransferase inhibitor 5’-azacytidine. Exposure of these cells to anti-neoplastic drugs for short periods of time (24-48 h) increased CAV1 expression through ROS production and MEK/ERK activation. In colon cancer cells, increased CAV1 expression enhanced migration and invasion *in vitro* via pathways requiring Src-family kinases, as well as Rac-1 activity. Finally, elevated CAV1 expression in colon cancer cells following exposure *in vitro* to sub-cytotoxic drug concentrations increased their metastatic potential *in vivo*. Therefore exposure of cancer cells to anti-neoplastic drugs at non-lethal drug concentrations induces signaling events and changes in transcription that favor CAV1-dependent migration, invasion and metastasis. Importantly, this may occur in the absence of selection for drug-resistance.

## INTRODUCTION

Caveolin-1 (CAV1) is an integral membrane protein that plays a dual role in tumor progression [1, 2]. On the one hand, CAV1 protein and mRNA levels are decreased in transformed fibroblasts and in breast and colon cancer cell lines. Also, CAV1 reduction in normal cells promotes cell transformation [3, 4]. Moreover, levels of CAV1 are reduced in several human tumors and, in these cells, CAV1 re-expression is often sufficient to block functions associated with the transformed cell phenotype [2, 5]. Available evidence indicates that CAV1 down-regulation, observed upon cell transformation, is due to CAV1 gene silencing by methylation of CG nucleotide enriched sequences (CpG islands) present in the *caveolin-1* promoter region. These CpG islands are not methylated in normal breast epithelial cells that express higher levels of CAV1; however in breast cancer cell lines that do not express CAV1, this region is highly methylated [6].

Alternatively, at later stages of cancer, CAV1 re-expression in lung adenocarcinoma, promotes filopodia formation and increases cell migration, as well as metastatic potential [7]. In breast cancer cell lines and melanomas, CAV1 expression favors focal adhesion turnover, cell migration and metastasis [8]. The increased levels of CAV1 have been associated with multidrug resistance (MDR) [9–11]. In HT29 colon cancer cells, resistance to the anti-neoplastic drug Methotrexate correlates with higher levels of CAV1 in comparison to untreated cells [1, 12] and silencing of CAV1 expression by RNA interference decreases the MDR phenotype and sensitizes cells to Methotrexate treatment [13].

Therefore, given the existing connection between elevated CAV1 expression and drug resistance, and the fact that different stress situations are known to augment CAV1 expression, we hypothesized that treatment with anti-neoplastic drugs at sub-cytotoxic doses may suffice to increase CAV1 levels in cancer cells where the *caveolin-1* gene was silenced epigenetically.

Cancer cells are known to possess higher levels of endogenous reactive oxygen species (ROS) than healthy cells, due to the decreased oxygen levels or hypoxia in the tumor microenvironment, enhanced cellular metabolism, mitochondrial dysfunction, increased growth factor receptor-mediated signaling and oncogene activity [14–18]. For this reason, one of the frequently employed strategies to kill cancer cells is by treatment with chemotherapeutic drugs that increase ROS levels beyond the adaptive threshold, which is lower in tumor cells than normal cells, thus triggering apoptosis [19–22]. However, the induction of ROS production by these chemotherapeutic agents can also activate proliferative and pro-survival signaling pathways, involving ERK1/2 and AKT. Activation of these pathways may favor the development of malignant characteristics, including increased cell migration associated with an elevated metastatic potential in cancer cells [23, 24]. Metastasis involves several steps, including degradation of the extracellular matrix, stromal invasion, intravasation to blood vessels, extravasation, migration and proliferation in other tissues and organs [25, 26] CAV1 interacts with proteins required for cell migration, such as β1 integrin [27] and filamin, promoting lamellipodia formation [28] and migration of fibroblasts in a RhoA-dependent fashion [29]. Alternatively, CAV1 promotes migration of metastatic breast cancer (MDA-MB-231), colon cancer (HT29(US)) and melanoma (B16F10, A375M) cells via activation of the Rab5-Rac1 signaling axis [2, 30, 31]. Increased CAV1 expression and particularly its phosphorylation on Y14 by Src family kinases are essential to enhance cell migration, invasion and anchorage-independent growth [32]. Importantly, in metastatic MDA-MB-231 cells, CAV1 is highly phosphorylated on Y14 in comparison with non-metastatic cancer cells [32] and in B16F10 melanoma cells CAV1 expression promotes matrix-specific migration, invasion and trans-endothelial migration in a Y14-dependent manner [33].

Given these attributes of CAV1, we evaluated the possibility that acute treatments of cancer cells with anti-neoplastic drugs could increase CAV1 expression. We also determined the signaling pathways involved in this up-regulation and the functional consequences of CAV1 re-expression in cancer cells were assessed using both *in vitro* and *in vivo* approaches. A DNA methylation inhibitor restored subdued basal CAV1 expression in colon and breast cancer cells. Additionally, Methotrexate and Etoposide decreased promoter region methylation, as well as increased CAV1 mRNA and protein levels in a MEK/ERK and ROS-dependent manner. Importantly, elevated CAV1 levels observed following drug exposure were associated with increased presence of tumor cells in ascites and metastasis *in vivo*. Finally, down-regulation of CAV1 using specific short hairpin RNA (shRNA) constructs sufficed to reverse these drug-induced effects, both *in vitro* and *in vivo*.

Taken together, these results implicate acutely enhanced CAV1 expression following the treatment with anti-neoplastic drugs at sub-cytotoxic concentrations in promoting the acquisition of malignant traits associated with a more aggressive cancer cell phenotype.

## RESULTS

### CAV1 expression is suppressed by methylation rather than histone acetylation in colon and breast cancer cell lines

**Figure 1:**
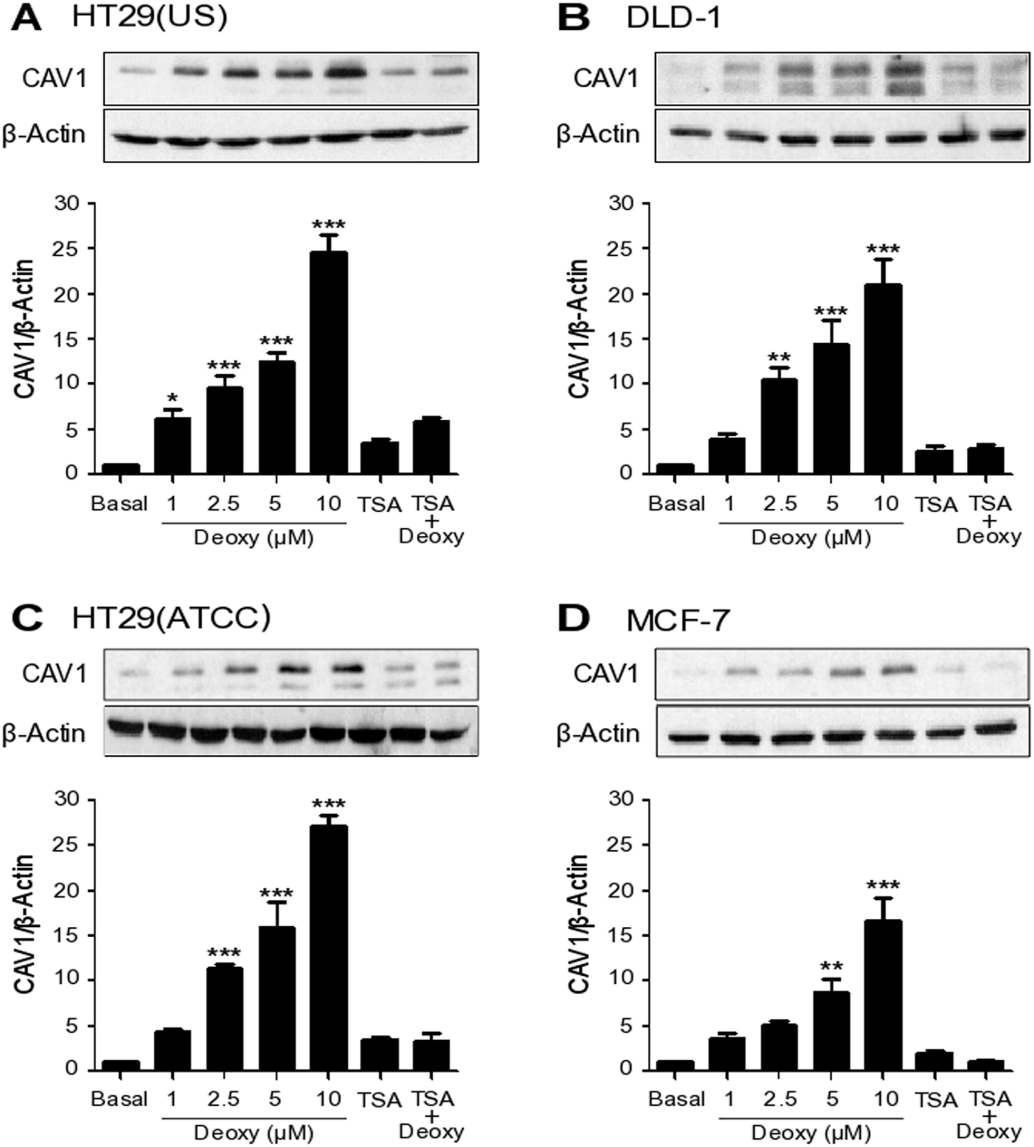
CAV1 expression is suppressed by DNA methylation in cancer cell lines Colon cancer cells **(A)** HT29(US), **(B)** DLD-1 and **(C)** HT29(ATCC) and breast cancer cells **(D)** MCF-7 were treated either with 5-aza dideoxycytidine (Deoxy, 1, 2.5, 5 or 10 μM) for 72 h, Trichostatin (TSA, 50 ng/ml) for 24 h or the combination of 2.5 μM Deoxy and 25 ng/ml TSA. Cells were harvested and total protein extracts were separated by SDS-PAGE (50 μg total protein per lane) and analyzed by Western blotting with antibodies against caveolin-1 (CAV1) and β-actin. The graphs show the expression of CAV1 normalized to β-actin (mean ± SEM) of 3 independent experiments. Significant differences in comparison with the untreated condition (Basal) are indicated ^***^ *p ≤ 0.001,* ^**^ *p ≤ 0.01,* ^*^ *p ≤ 0.05*.

The 5’ region of the *caveolin-1* promoter is enriched in CpG islands that are methylated in breast, lung, ovarian and colon cancer cell lines [5, 6, 34, 35]. We have previously shown in colon cancer cell lines that increased CAV1 expression arises in conjunction with the development of drug-resistance [2]. At least, two potential hypotheses may be entertained to explain these observations. One is that exposure to chemotherapeutic drugs selects for cells that are drug-resistant and that these express higher CAV1 levels. Alternatively, the drugs might directly induce CAV1 expression by mechanisms that remain to be determined and expression of CAV1 in this context would favor the development of a more malignant phenotype. To distinguish between these possibilities, we first evaluated whether the low levels of basal CAV1 expression observed in the colon cancer cells (HT29(US), DLD-1 and HT9(ATCC)) and in the breast cancer cell line MCF7 were attributable to promoter methylation. Treatment of these cells with 5-aza dideoxycytidine (Deoxy, 5 μM), a DNA methylation inhibitor, increased CAV1 expression both in colon (Figure 1A–1C) and breast cancer cell lines (Figure 1D). Conversely, treatment with the histone deacetylase (HDAC) inhibitor, Trichostatin (50 ng/ml), did not significantly increase CAV1 expression and appeared to block the increase due to Deoxy instead (Figure 1). These observations indicate that basal expression of CAV1 is suppressed both in colon and breast cancer cells by DNA methylation rather than histone deacetylation.

### Anti-neoplastic drugs increase CAV1 expression in colon and breast cancer cell lines

**Figure 2:**
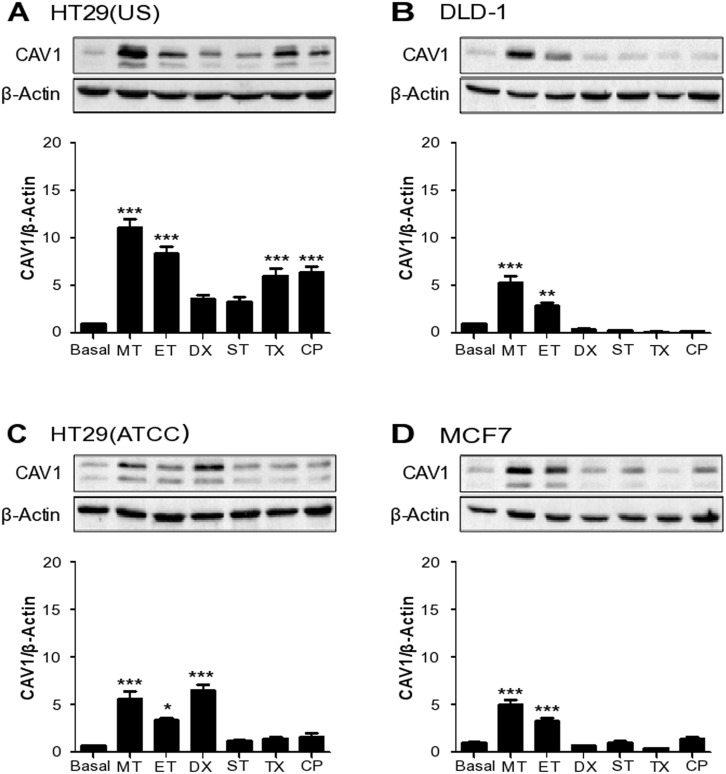
Anti-neoplastic drugs increase CAV1 expression in colon and breast cancer cell lines Colon cancer cells **(A)** HT29(US), **(B)** DLD-1, **(C)** HT29(ATCC) and breast cancer cells **(D)** MCF-7 were treated with 100 nM Methotrexate (MT), 10 μM Etoposide (ET), 1 μM Doxorubicin (DX), 5 nM Staurosporine (ST), 5 nM Taxol (TX) or 100 nM Cisplatin (CP) for 24 h. Cells were harvested and total protein extracts were separated by SDS-PAGE (50 μg total protein per lane) and analyzed by Western blotting with antibodies against caveolin-1 (CAV1) and β-actin. The graphs show the expression of CAV1 normalized to β-actin (mean ± SEM) of 3 independent experiments. Significant differences in comparison with the untreated condition (Basal) are indicated ^***^ *p ≤ 0.001,* ^**^ *p ≤ 0.01,* ^*^ *p ≤ 0.05*.

CAV1 expression increases as tumors progress and is associated there with increased metastasis [1, 7, 36] and MDR [1, 2, 9, 11]. These observations, together with others showing that CAV1 expression is frequently up-regulated in response to stress situations [37–39], caught our attention. Given this CAV1 connection to a more aggressive phenotype at later stages of cancer disease, we reasoned that treatment with cytotoxic drugs using conditions that may not induce cell death might in fact promote CAV1 expression and thereby favor cancer progression. With this in mind, we treated colon and breast cancer cell lines with different chemotherapeutic agents that are routinely employed in cancer therapy, namely Methotrexate, Etoposide, Doxorubicin, Staurosporine, Taxol and Cisplatin. Note that for all the drugs employed, with the exception of Doxorubicin, cell viability was around 50-90% at the end of the treatment (Supplementary Table 1). We next evaluated CAV1 protein expression in the cancer cells. Subsequently, optimal conditions for CAV1 expression were defined by treating HT29(US) cells either with increasing concentrations for a specific period of time (Supplementary Figure 1) or a given concentration for varying periods of time (Supplementary Figure 2). All the anti-neoplastic drugs employed, increased CAV1 expression in the colon cancer cell line HT29(US), but the effect was stronger with the dihydrofolate reductase inhibitor Methotrexate, and the topoisomerase II inhibitor Etoposide (Figure 2A, Supplementary Figure 1A and 1B). In the case of DLD-1 cells, Methotrexate and Etoposide were the only two drugs that significantly increased CAV1 protein levels (Figure 2B). This was also the case for the breast cancer cell line MCF7 (Figure 2D), while for HT29(ATCC) cells, besides Methotrexate and Etoposide, Doxorubicin also increased CAV1 expression (Figure 2C).

Considering that the chemotherapeutic agents Methotrexate and Etoposide increased CAV1 expression in all of the cell models evaluated here, we decided to utilize these two agents to induce CAV1 and to identify the mechanism(s) by which this increase occurs.

**Figure 3:**
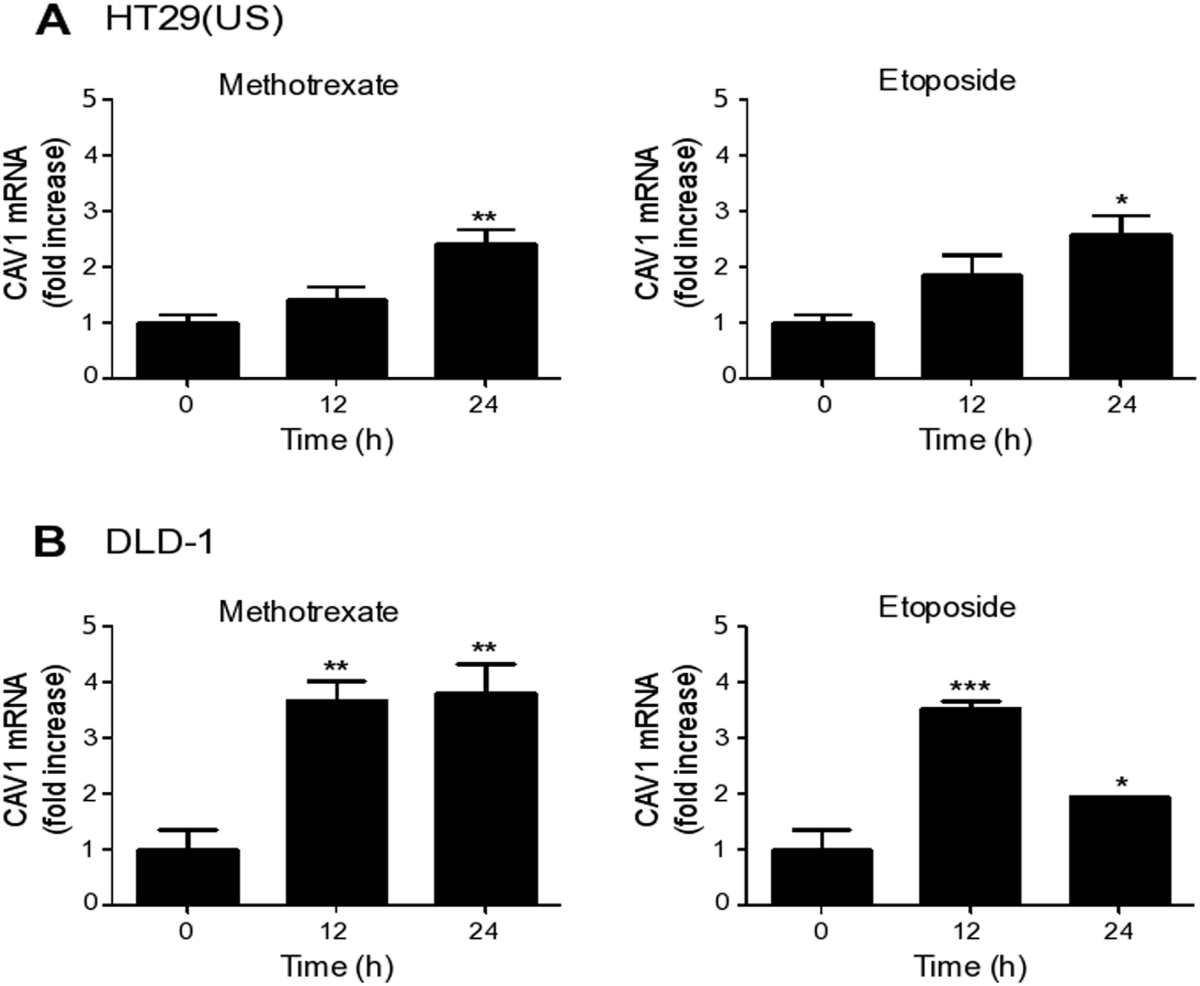
Methotrexate and Etoposide induce an increase in CAV1 mRNA levels in colon cancer cell lines Colon cancer cells **(A)** HT29(US) and **(B)** DLD-1 were treated with 100 nM Methotrexate or 10 μM Etoposide for 12 and 24 h. CAV1 mRNA levels were evaluated by quantitative RT-PCR analysis, using β-actin as an internal control. Values obtained by analysis of three independent experiments are shown for CAV1 mRNA following standardization to β-actin (mean ± SEM) and after normalizing to the values obtained for untreated (0 h) samples. Statistically significant differences compared with the controls (time 0) are indicated ^***^p ≤ 0.001, ^**^p ≤ 0.01, ^*^p ≤ 0.05.

As a first step, we determined whether up-regulation of CAV1 by Methotrexate or Etoposide occurred at the transcriptional level. To that end, we treated the colon cancer cell lines HT29(US) and DLD-1 with either Methotrexate or Etoposide for 12 and 24 h, and evaluated CAV1 mRNA levels by qPCR analysis. Already after 12 h of treatment, we observed an increase in CAV1 mRNA levels in HT29(US) (Figure 3A) and DLD-1 cells (Figure 3B). Thus, the anti-neoplastic drugs Methotrexate and Etoposide likely increase CAV1 protein levels by a transcriptional mechanism following promoter region demethylation.

### Methotrexate and Etoposide induce CAV1 promoter demethylation in colon cancer cells

**Figure 4:**
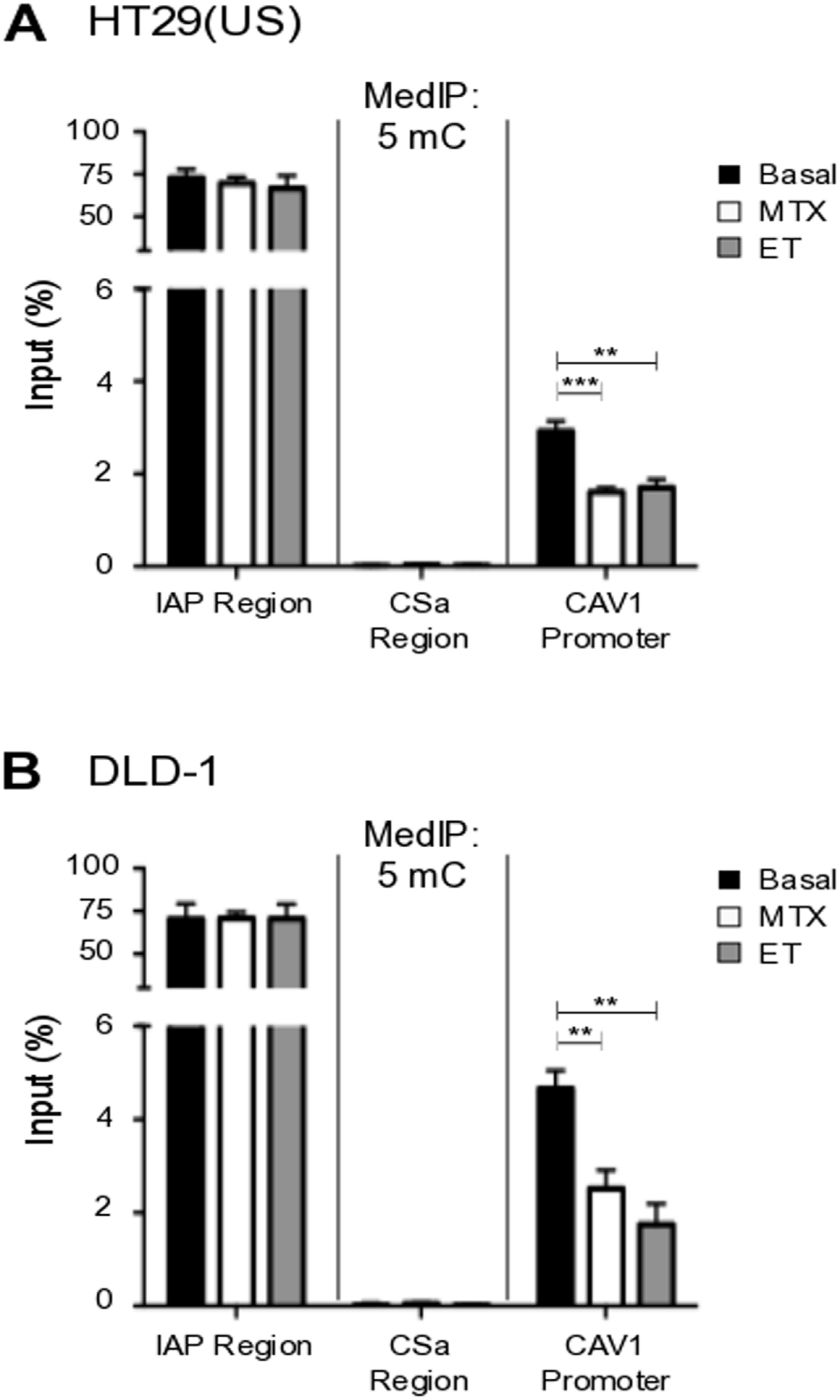
Methotrexate and Etoposide induce CAV1 promoter demethylation in colon cancer cells **(A)** HT29(US) and **(B)** DLD-1 colon cancer cells were treated with 100 nM Methotrexate (MT) or 10 μM Etoposide (ET) for 48 h. Genomic DNA (gDNA) was denatured for 10 min at 95°C and then immunoprecipitated using the anti-5-methylcytidine antibody (5mC). Affinity purified DNA was then evaluated using qPCR analysis, defining the enrichment levels as a percentage of the input material. Specific primers were used to analyze the CAV1 proximal promoter (CAV1 promoter) or negative (CSa region) and positive control regions (IAP region). Statistically significant differences compared with the control (Basal, black bars) are indicated ^***^p ≤ 0.001, ^**^p ≤ 0.01.

To demonstrate that increased CAV1 expression following treatment with anti-neoplastic drugs was indeed associated with demethylation of the CAV1 promoter region, the extent of methylated DNA was determined in HT29(US) (Figure 4A) and DLD-1 (Figure 4B) cells after treatment for 48 h with Methotrexate or Etoposide using chromatin immunoprecipitation followed by qPCR. In both colon cancer cell lines, treatment with the anti-neoplastic drugs decreased significantly methylation of the CAV1 promoter region (Figure 4), which likely explains, at least in part, the increase in CAV1 mRNA (Figure 3) and protein (Figure 2) levels observed following drug exposure.

### CAV1 up-regulation requires ERK signaling and ROS production

**Figure 5:**
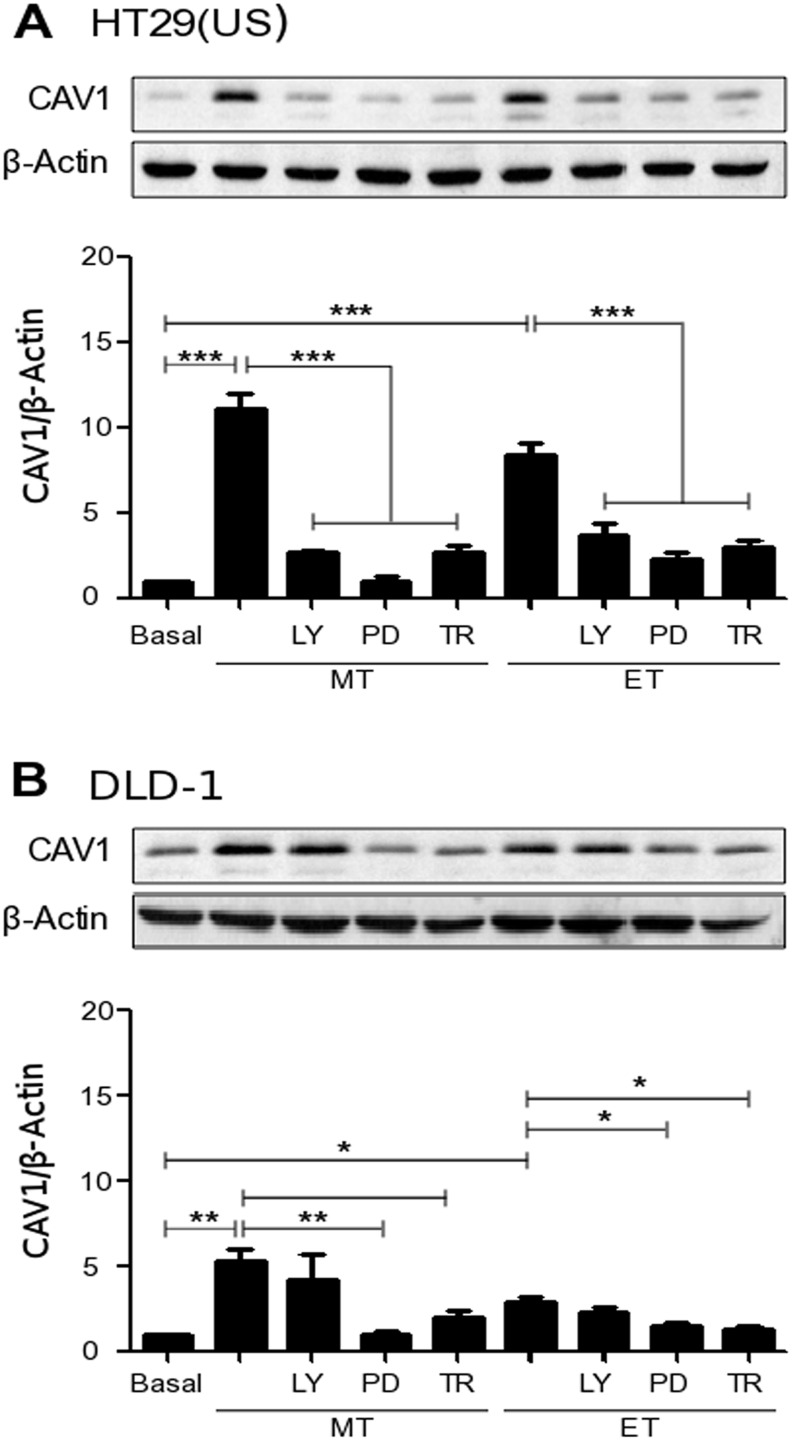
Effects of either PI3K or MEK inhibition as well as the antioxidant Trolox on the up-regulation of CAV1 induced by Methotrexate and Etoposide Colon cancer **(A)** HT29(US) and **(B)** DLD-1 cells were treated with either the PI3K inhibitor LY294002 (LY, 10 μM), the MEK inhibitor PD98059 (PD, 50 μM) or the vitamin E analog, Trolox (TR, 2 mM) for 30 min before treatment with 100 nM Methotrexate (MT) or 10 μM Etoposide (ET) for 48 h. Cells were harvested and total protein extracts were separated by SDS-PAGE (50 μg total protein per lane) and analyzed by Western blotting with antibodies against CAV1 and β-actin. The graphs show the expression of CAV1 normalized to β-actin (mean ± SEM) averaged from 3 independent experiments. Significant differences in comparison with the untreated condition (Basal) are indicated ^***^p ≤ 0.001, ^**^p ≤ 0.05, ^*^p ≤ 0.01.

In order to study the signaling pathways involved in CAV1 up-regulation, induced by anti-neoplastic drugs, cancer cells were treated with either the PI3K inhibitor LY294002 (10 μM), the MEK inhibitor PD98059 (50 μM) or with the antioxidant Trolox (2 mM), prior to addition of Methotrexate or Etoposide. Inhibition of MEK and pre-treatment with Trolox, both individually precluded CAV1 up-regulation induced by either Methotrexate or Etoposide in colon cancer cell lines HT29(US) (Figure 5A), DLD-1 (Figure 5B) and HT29(ATCC) (Supplementary Figure 3A), as well as in MCF7 breast cancer cells (Supplementary Figure 3B). LY294002 pre-treatment inhibited CAV1 up-regulation induced by Methotrexate in HT29(US) cells (Figure 5A), and by Etoposide in MCF7 cells (Supplementary Figure 3B), but not in any of the other cases. These results suggested that MEK/ERK activation and ROS production represent the predominant pathways involved in CAV1 re-expression induced by anti-neoplastic drugs in cancer cells.

Subsequently, we evaluated whether these anti-neoplastic drugs activate ERK in DLD-1 colon cancer cells, using phospho-ERK specific antibodies. Methotrexate treatment induced three ERK 1/2-phosphorylation peaks, at 5 and 30 min and then again after 6-10 h of exposure (Supplementary Figure 4A). Alternatively, Etoposide induced two ERK 1/2-phosphorylation peaks, at 5 min and after 15 h of drug exposure (Supplementary Figure 4B). Considering that Methotrexate and Etoposide induced an increase in CAV1 mRNA after 12 h of treatment, the findings suggest that ERK1/2 activation by Methotrexate and the first peak of ERK1/2 activation by Etoposide are likely linked to transcriptional upregulation of CAV1.

By flow cytometry we then observed that Methotrexate induced a mild increase in intracellular ROS levels at 20 h in HT29(US) (Supplementary Figure 5A, black bars) and a more marked increase in DLD-1 cells (Supplementary Figure 5B, black bars), whereas Etoposide increased ROS levels starting at 20 h of exposure in HT29(US) cells (Supplementary Figure 5A, white bars) and after 14 h in DLD-1 cells (Supplementary Figure 5B, white bars).

**Figure 6:**
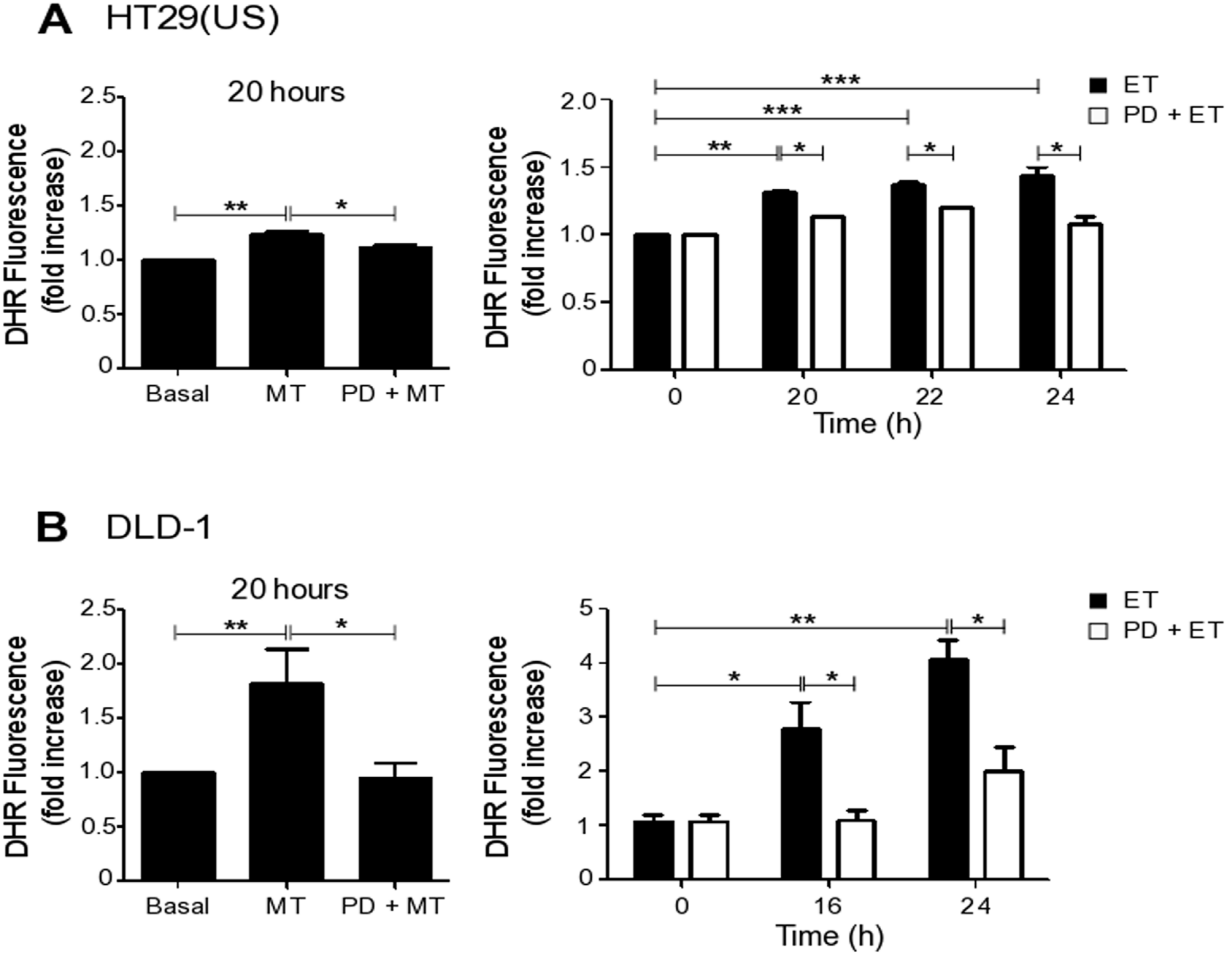
MEK inhibition reduces ROS production induced by Methotrexate and Etoposide **(A)** HT29(US) or **(B)** DLD-1 colon cancer cells (3 x 10^6^) were seeded in 24-well plates and, after 24 h, were treated with the MEK inhibitor PD98059 (PD, 50 μM) for 30 min, added prior to treatment with 100 nM Methotrexate for 20 h or 10 μM Etoposide for the indicated time periods. Cells were washed 3 times with PBS and subsequently incubated with trypsin for 5 min. Once in suspension, cells were loaded with the probe DHR123 (1.4 μg/ml) in RPMI media without serum for 30 min and then the reaction was stopped on ice. The extent of DHR123 oxidation was determined by flow cytometry. The graphs show DHR123 fluorescence normalized to the untreated condition (Basal) (mean ± SEM) averaged from 3 independent experiments. Significant differences are indicated ^***^p≤ 0.001, ^**^p ≤ 0.01, ^*^p ≤ 0.05.

To determine whether ERK1/2 activation resides upstream of ROS formation or vice-versa, we treated colon cancer cells with the MEK inhibitor PD98059 prior to Methotrexate or Etoposide exposure. Inhibition of the MEK/ERK pathway reduced ROS induced by both Methotrexate and Etoposide in HT29(US) (Figure 6A) and in DLD-1 (Figure 6B) cell lines. Thus, MEK/ERK activation lies upstream of ROS formation, and both are required for drug-induced up-regulation of CAV1 transcription (Figure 5).

### CAV1 up-regulation induced by Methotrexate and Etoposide enhances cancer cell migration

**Figure 7:**
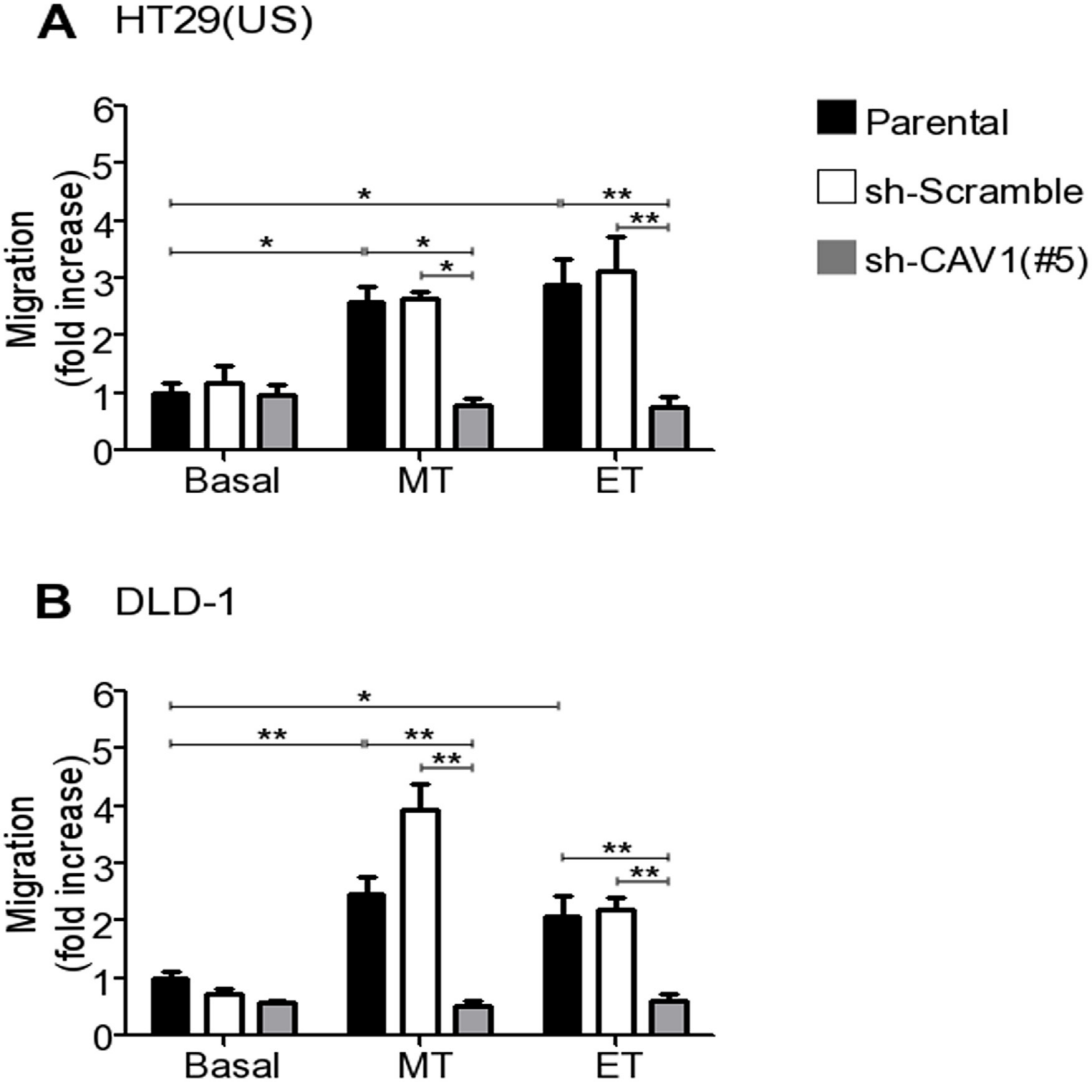
CAV1 silencing precludes the increase in cell migration induced by Methotrexate and Etoposide in colon cancer cell lines Parental, sh-Scramble and sh-CAV1 (#5) **(A)** HT29(US) or **(B)** DLD-1 cells (6 x 10^5^) were seeded in 6 cm plates 24 h before treatment with 100 nM Methotrexate or 10 μM Etoposide for 48 h. Cells (2 x 10^5^) were then seeded in Boyden chambers coated with fibronectin (2 μg/ml) on the lower side and allowed to migrate for 7 h (HT29(US) cells) or 5 h (DLD1 cells). The cells that migrated through the pores were stained and counted. Values obtained were normalized to those obtained for parental cells without treatment. The graphs show the averages of values from 3 independent experiments (mean ± SEM). Significant differences are indicated ^**^p ≤ 0.01, ^*^p ≤ 0.05.

CAV1 up-regulation has been linked to an increase in the migration of breast, colon, melanoma [12, 31] and endometrial cancer cells [39]. To evaluate the functional consequences of CAV1 re-expression induced by anti-neoplastic drugs, we silenced CAV1 with a specific shRNA (sh-Cav-1 (#5)) in HT29(US) and DLD-1 cells (Supplementary Figure 6A and 6B, respectively). Parental colon cancer cells or control shRNA (sh-Scramble) cells treated with Methotrexate or Etoposide migrated approximately 3 times more rapidly than untreated cells (Figure 7A and 7B). However, the increase in cell migration induced by anti-neoplastic drugs, was precluded by CAV1 silencing in HT29(US) (Figure 7A, grey bars) and DLD-1 (Figure 7B, grey bars) cells. Hence, Methotrexate- and Etoposide-enhanced cell migration depends on CAV1 re-expression in HT29(US) and DLD-1 colon cancer cells.

### Anti-neoplastic drugs increase cell migration in a MEK/ERK, Src kinase and ROS dependent manner

**Figure 8:**
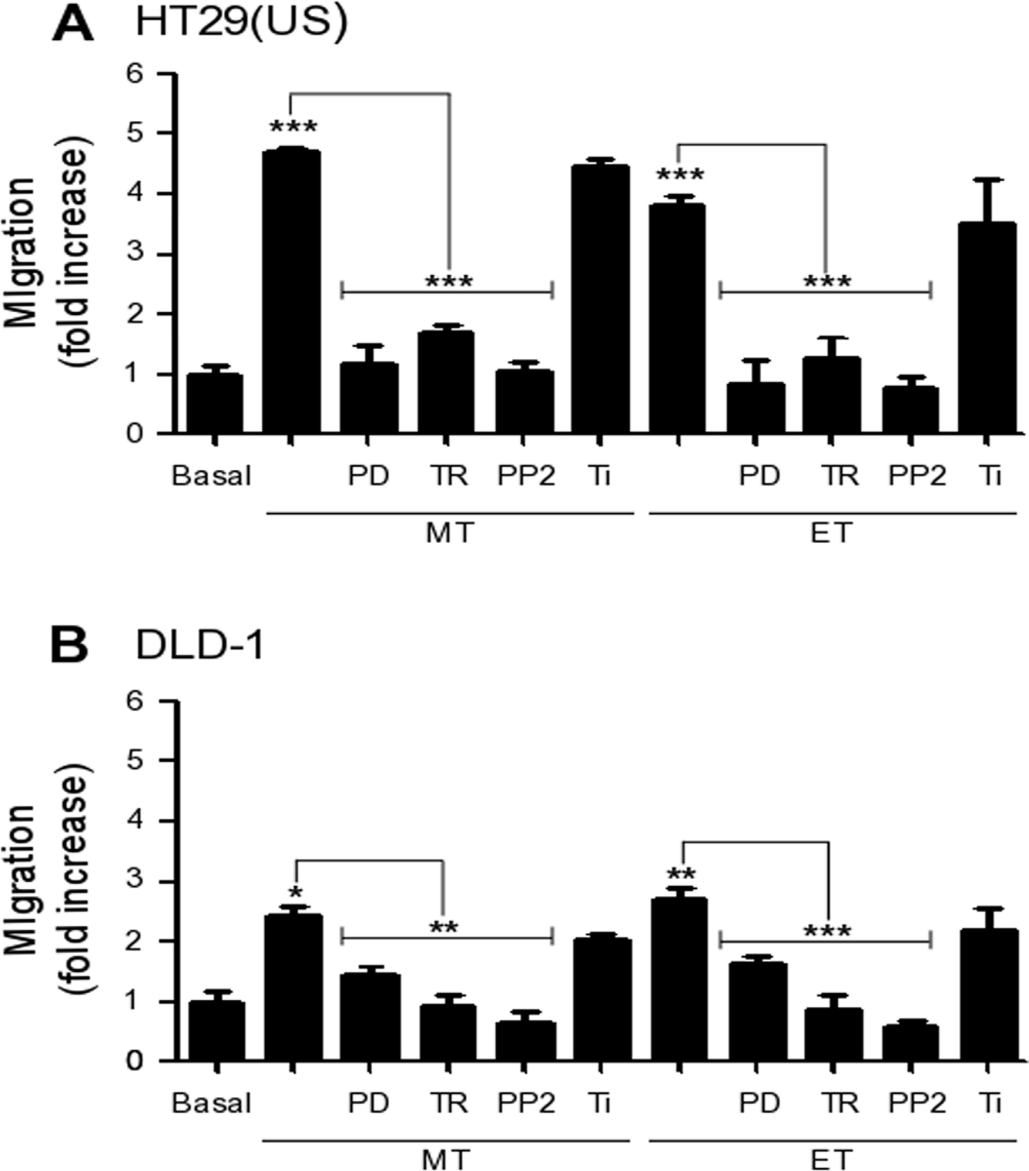
Effect of MEK and Src family kinase inhibition, as well as the anti-oxidants Trolox and Tiron on cell migration induced by Methotrexate and Etoposide **(A)** HT29(US) or **(B)** DLD-1 cells (6 x 10^5^) were seeded in 6 cm plates 24 h before treatment with the MEK inhibitor PD98059 (PD, 50 μM), the Src family kinase inhibitor PP2 (1 mM), the vitamin E analog Trolox (TR, 2 mM) or the superoxide scavenger Tiron (Ti, 4 mM) for 30 min (PD, TR and Ti) or 1 h (PP2) before treatment with 100 nM Methotrexate or 10 μM Etoposide for 48 h. Cells (2 x 10^5^) were then seeded in Boyden chambers coated with fibronectin (2 μg/ml) and allowed to migrate for 7 h (HT29(US) cells) or 5 h (DLD1 cells). The cells that migrated through the pores were stained and counted. Values were normalized to those obtained for cells without treatment (Basal). The graphs show the averages of results from 3 independent experiments (mean ± SEM). Significant differences are indicated ^***^p ≤ 0.001, ^**^p ≤ 0.01, ^*^p ≤ 0.05.

To determine whether the aforementioned signaling pathways responsible for increasing CAV1 expression were also responsible for the increase in cell migration, we pre-treated HT29(US) and DLD-1 cells with either the MEK inhibitor PD98058 or the antioxidants Trolox or Tiron. As anticipated, MEK/ERK inhibition precluded the increase in cell migration induced by Methotrexate or Etoposide in HT29(US) (Figure 8A) and DLD-1 (Figure 8B) cells. Likewise, we observed the same inhibitory effects in Trolox pre-treated cells; however pre-treatment with the superoxide scavenger Tiron, did not affect either Methotrexate or Etoposide enhanced cell migration. Because Trolox is a membrane-bound vitamin E-analog, these results suggest that ROS generation at the plasma membrane is likely to be responsible for favoring cell migration.

Previous studies showed that CAV1 phosphorylation on tyrosine 14 is required to promote CAV1-dependent cell migration [12] and that Src family kinases are activated by ROS [15]. Thus, we treated cells with Methotrexate or Etoposide. As anticipated, PP2 pre-treatment blocked the increase in cell migration observed in both cells lines following treatment with either Methotrexate or Etoposide (Figure 8), suggesting an important role for CAV1 phosphorylation on tyrosine 14 in cell migration induced by anti-neoplastic drugs.

### Methotrexate and Etoposide enhance metalloproteinase activity via CAV1 up-regulation

**Figure 9:**
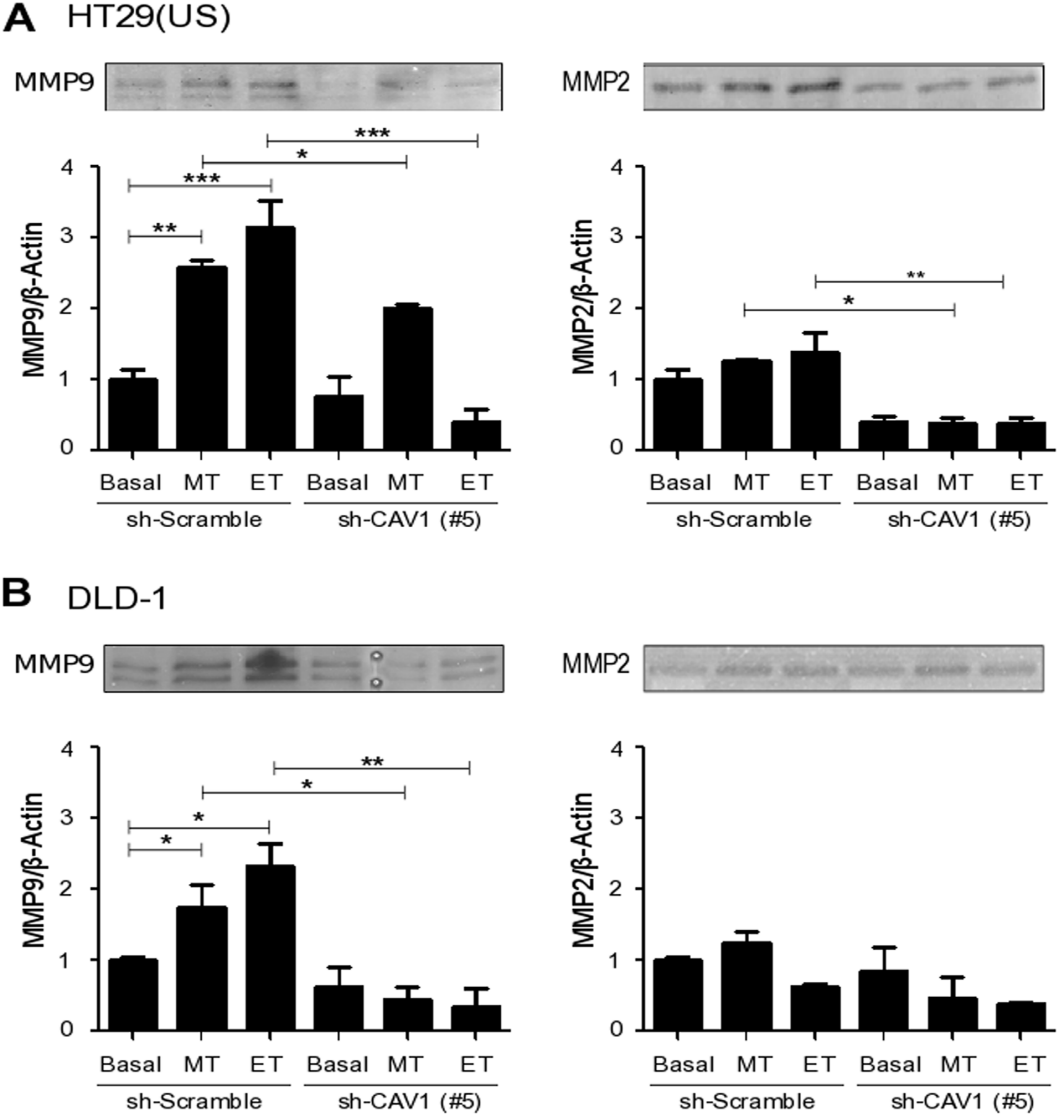
CAV1 silencing decreases metalloproteinase activity induced by Methotrexate and Etoposide in colon cancer cell lines sh-Scramble and sh-CAV1 (#5) **(A)** HT29(US) or **(B)** DLD1 cells (6 X 10^5^) were seeded in 6 cm plates 24 h before treatment with 100 nM Methotrexate or 10 μM Etoposide for 48 h. Cells were harvested and total protein extracts were subjected to gelatin zymography. The graphs show the densitometric analysis of gelatinolytic activity detected at 92 kDa (pro-MMP9) and 72 kDa (pro-MMP2) averaged from 3 independent experiments (mean ± SEM). Significant differences are indicated, ^***^p≤ 0.001, ^**^p ≤ 0.01, ^*^p ≤ 0.05.

Metastasis is a complex process that involves degradation of the extracellular matrix, invasion of the stromal tissue, intravasation into the circulation, extravasation, migration and proliferation in other tissues and organs [25, 26]. The gelatinases B, MMP9 and MMP2 are members of the matrix metalloproteinase family that play a critical role in cell invasion and metastasis. Thus, we next investigated whether Methotrexate and Etoposide treatments modulated metalloproteinase activity. Indeed, both Methotrexate and Etoposide exposure increased MMP9 activity in HT29(US) (Figure 9A) and DLD-1 (Figure 9B) cells in comparison with non-treated cells. For MMP2 activity, a very modest increase was only detected in HT29(US) cells. Importantly, in both HT29(US) and DLD-1 cells, activation of MMP9 and MMP2 was reduced in cells where CAV1 expression had been silenced, in agreement with data reported in the literature linking CAV1 expression to the transcriptional upregulation of metalloproteinases in vascular smooth muscle cells [40] and cancer cells [41]. For MMP2, CAV1 silencing lead to a significant decrease in basal activity in HT29(US) cells (Figure 9).

### Anti-neoplastic drugs increase cancer cell invasion in a manner dependent on Src family kinases and Rac1 activation

**Figure 10:**
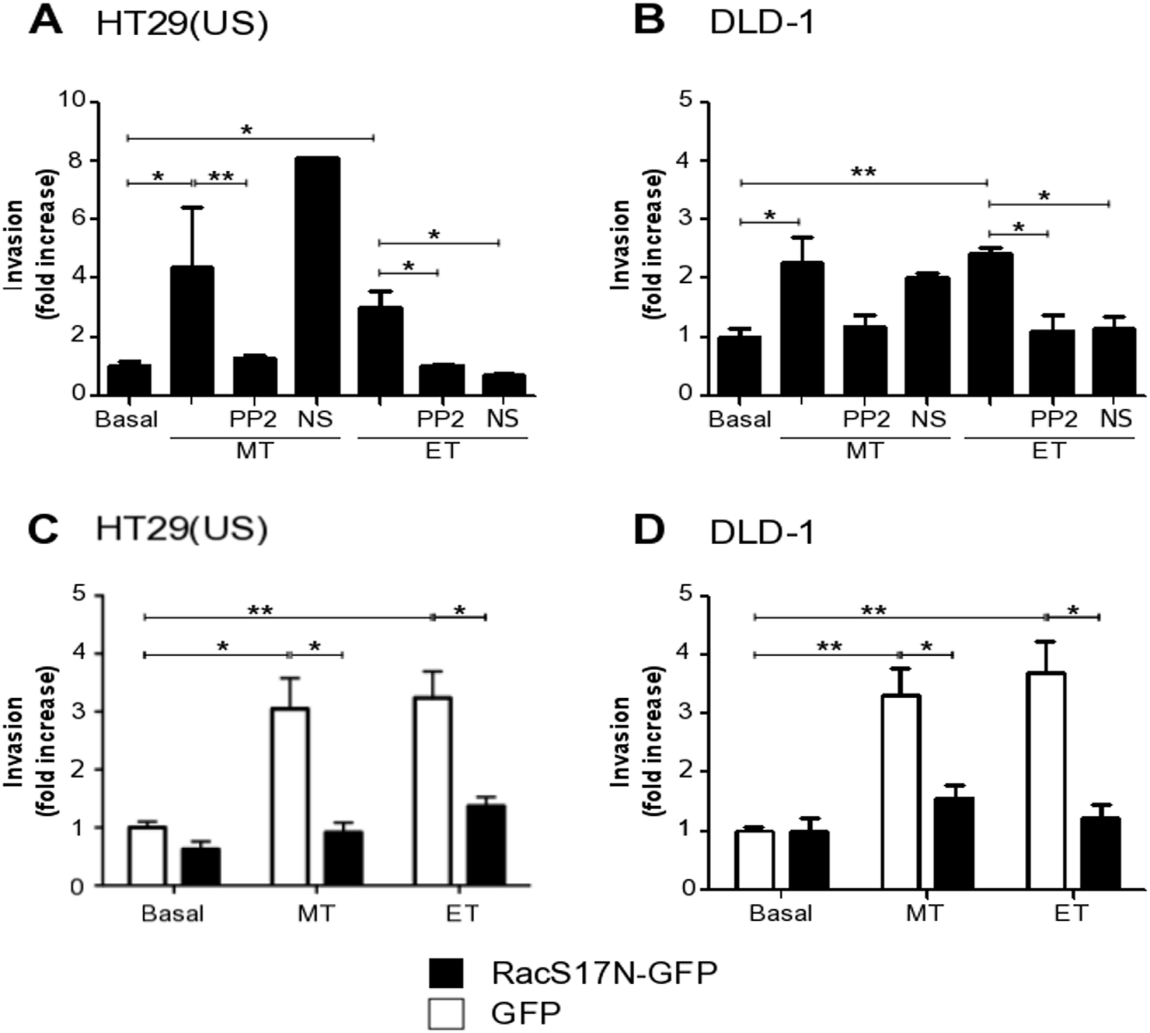
Methotrexate and Etoposide increase cancer cell invasion in a Src family kinase and Rac1 dependent manner **(A)** HT29(US) or **(B)** DLD1 (6 X 10^5^) cells were seeded in 6 cm plates 24 h before pre-treatment with either the Src family kinase inhibitor, PP2 (1 mM) or with the Tiam1 inhibitor, NS (NSC 23766, 100 mM) for 60 min followed by the treatment with 100 nM Methotrexate (MT) or 10 μM Etoposide (ET) for 48 h. **(C)** HT29(US) or **(D)** DLD1 (6 X 10^5^) cells were transfected with GFP (white bars) or with the Rac1 dominant-negative, RacS17N (black bars), 24 h before the treatment with 100 mM Methotrexate (MT) or 10 μM Etoposide (ET) for 48 h. Then, cells (2 x 10^5^) were seeded in Matrigel-coated chambers and allowed to invade the matrix for 24 h. The cells that accumulated on the lower surface of the membrane were then stained and counted. Values obtained were normalized to those obtained for cells without treatment (Basal). The graphs show the averages of results from 3 independent experiments (mean ± SEM). Significant differences are indicated, ^**^p ≤ 0.01, ^*^p ≤ 0.05.

Because Methotrexate and Etoposide increased metalloproteinase activity, we investigated whether the treatment with anti-neoplastic drugs induced colon cancer cell invasion. As expected, Methotrexate and Etoposide enhanced invasion of HT29(US) (Figure 10A) and DLD-1 cells (Figure 10B) in a manner dependent on Src family kinase activity, since pre-treatment with the Src family kinase inhibitor PP2 impaired cell invasion in both cases (Figure 10A and 10B).

In melanoma cells, CAV1 expression induces an increase in cell migration and invasion by the activation of Rac1 [31]. Based on these findings, we investigated in our models whether Rac1 activity was implicated in invasion triggered by treatment with the anti-neoplastic drugs. To that end, we transfected HT29(US) and DLD-1 cells with a Rac1 dominant-negative (RacS17N-GFP) and observed that Rac1 activity was necessary for the increase in invasion of these cells induced by Methotrexate and Etoposide (Figure 10C and 10D). Tiam1, a GEF implicated in Rac1 activation downstream of CAV1 is inhibited by the compound NSC23766 [31]. As anticipated, pre-treatment with NSC23766 also blocked Etoposide-induced invasion by colon cancer cells (Figure 10A and 10B). Surprisingly, however, this inhibitor did not reduce Methotrexate-induced invasion of either HT29(US) or DLD-1 cells (Figure 10A and 10B). Thus, a Tiam1-independent, but Rac1-dependent, pathway is likely to participate in CAV1-enhanced invasion following Methotrexate treatment.

### Treatment with anti-neoplastic drugs increases cancer cell metastasis *in vivo*

**Figure 11:**
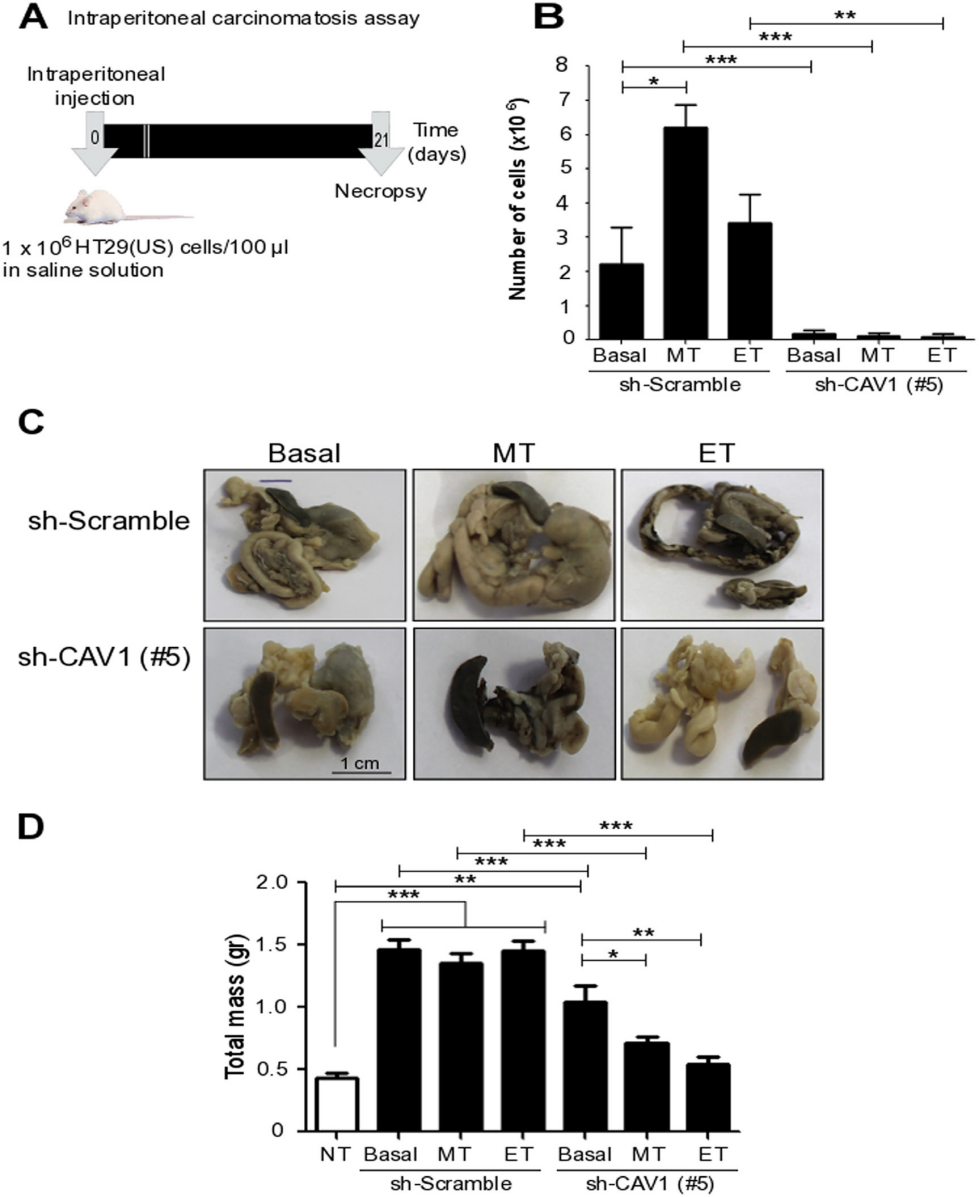
*Ex vivo* treatment of colon carcinoma cells with Methotrexate and Etoposide increases the number of CAV1-expressing cells in ascites fluid and metastasis *in vivo* **(A)** Schematic summarizing events in the intraperitoneal carcinomatosis assay: Seven week-old BalbC/NoD/SciD mice (5 mice per group) were injected intraperitoneally with 1 x 10^6^ sh-Scramble or sh-CAV1 (#5) HT29(US) cells treated with 100 nM Methotrexate (MT) or 10 μM Etoposide (ET) for 48 h prior to injection. After 21 days, the animals were euthanized and paracentesis was analyzed. **(B)** The graph shows the number of viable cells in the ascitic fluid in each condition. **(C)** Representative images at 21 days post-cell injection showing the intestine, pancreas, spleen and stomach of mice injected with HT29(US) sh-Scramble (upper panel) or HT29(US) sh-CAV1 (#5) (lower panel) that prior to injection were either not treated (Basal) or treated with 100 nM Methotrexate (MT) or 10 μM Etoposide (ET) for 48 h. **(D)** The graph shows the total mass of the solid tumors and the infiltrated organs (intestine, pancreas, spleen and stomach) of mice injected with HT29(US) sh-Scramble or HT29(US) sh-CAV1 (#5) that were previously either not treated (Basal) or treated with 100 nM Methotrexate (MT) or 10 μM Etoposide (ET) for 48 h, as well as the total weight of the organs in non-treated mice (NT). Significant differences are indicated, ^***^p ≤ 0.001, ^**^p ≤ 0.01, ^*^p ≤ 0.05.

Finally, to underscore the importance of these observations, we explored the effects of acute Methotrexate and Etoposide treatments on the metastatic properties of colon cancer cells *in vivo*. To this end we used a murine model of intraperitoneal carcinomatosis, in order to evaluate intra-abdominal dissemination of malignant cells (Figure 11) [42]. HT29(US) human colon cancer cells transduced with either (sh-Scramble) or (sh-Cav1(#5)) containing lentivirus were treated for 48 h either with Methotrexate or Etoposide, washed and then injected intraperitoneally (1x10^6^) into BalbC/NoD/SciD mice. After 12 days, the mice injected with HT29(US)(sh-Scramble) cells, which were treated with either Methotrexate or Etoposide showed an increase in the Morton punctuation [43, 44]. After 21 days, the mice were euthanized and the extent of paracentesis was determined by counting the number of live cells in the intraperitoneal ascitic fluid. Under basal conditions (non-treated cells), in mice injected with CAV1-expressing HT29(US) cells (HT29(US) sh-Scramble), the ascites fluid contained more cells than observed for mice injected with CAV1-silenced cells (HT29(US) sh-CAV1 (#5)) (Figure 11B). Furthermore, in mice that were injected with HT29(US) sh-Scramble cells after treatment with either Methotrexate or Etoposide, the malignant ascites volume was increased (data not shown) in comparison with either non-treated cells or cells that did not express CAV1 (HT29(US) sh-CAV1 (#5)) (Figure 11B).

Also the solid tumor mass was evaluated for each condition. For mice injected with HT29(US) cells expressing CAV1 (HT29(US) sh-Scramble), treated or not with either Methotrexate or Etoposide, the tumor mass associated with the intestine, pancreas, spleen and stomach was higher than the tumor mass generated after injection of cells that did not express CAV1 (HT29(US) sh-CAV1 (#5)) (Figure 11C and 11D). Moreover, in cells that did not express CAV1, treatment with these anti-neoplastic drugs produced smaller tumors than mice injected with non-treated HT29(US) sh-CAV1 (#5) cells (Figure 11C and 11D). These results suggest that enhanced expression of CAV1 observed in carcinoma cells following exposure to chemotherapeutic drugs is necessary and sufficient to promote tumor cell metastasis.

## DISCUSSION

CAV1 plays a dual role in the development of cancer. At early stages it is considered a tumor suppressor and at later stages it contributes to a more aggressive phenotype [1]. In part, CAV1 does so by modulating drug pump expression and function [45]. This requires extensive periods of exposure to drugs. For instance, incubation of HT29(US) cells with increasing concentrations of Etoposide (50 – 1000 nM) gradually increased CAV1 levels over extended periods of time (approx. 3-6 months) and this increase correlated with enhanced multi-drug resistance (Montoya and Quest, unpublished data). These observations are consistent with the classic view that malignant cancer cell traits are acquired as a consequence of a long-term selection process. However, the findings presented here point towards the existence of mechanisms by which short-term (24-48 h exposure) exposure to anti-neoplastic drugs induces changes particularly in CAV1 expression that favor the acquisition of a more aggressive, metastatic phenotype. These findings are consistent with predictions made by the phenotype-switching model suggesting that rapid changes in cancer cell behavior can be triggered by “environmental” factors [46].

Chemotherapy remains an important and effective treatment for early stage cancer; however, resistance to conventional chemotherapeutic agents poses a tremendous challenge, and represents a major obstacle in the treatment of cancer, which may potentially lead to tumor relapse and failure of the therapy [47, 48]. The mechanisms contributing to drug resistance either reduce effective intracellular drug concentration and/or reduce sensitivity to the drugs. Here we show that the treatment with several anti-neoplastic drugs at generally sub-cytotoxic concentrations induced up-regulation of CAV1. For Methotrexate and Etoposide, we further show that this increase is associated with enhanced cell migration, invasion and metastasis, and therefore, with a more malignant phenotype.

Two major types of epigenetic alterations closely linked to cancer are aberrant DNA methylation and covalent histone modifications [49]. The 5’ region of the *caveolin-1* promoter is enriched in CpG islands that are not methylated in normal breast epithelial cells and express higher levels of CAV1; however, in breast cancer cell lines that do not express CAV1, this region is highly methylated [6]. The methylation pattern of the CAV1 promoter also changes according to the level of malignancy and metastatic potential in breast cancer tumors [50]. In HT29 colon cancer cells, CAV1 expression is silenced by treatment of the cells with butyrate, which induces histone hyperacetylation among other effects [51]. Thus both modes of regulation have been implicated in CAV1 silencing in cancer cells. Here we show that suppression of basal CAV1 expression in the colon and breast cancer cells employed in our studies is reverted by inhibition of DNA methylation using 5-aza dideoxycytidine. Also, we provide direct evidence in DNA immunoprecipitation assays using methylation-specific antibodies that the *CAV1* promoter region is demethylated upon exposure of colon cancer cells to Methotrexate or Etoposide.

An important objective of the present study was to show that although these drugs elicit their cytotoxic effects via distinct mechanisms when engaging tumor cells, they increased the expression of CAV1 even at sub-cytotoxic concentrations by triggering signaling pathways commonly associated with tumor survival and progression. Indeed, consistent with this view, induction of CAV1 expression by either Methotrexate or Etoposide was very effectively blocked by the MEK inhibitor (PD98059) and the anti-oxidant Trolox in both HT29(US) and DLD-1 cells. However, the PI3K inhibitor LY294002, was only effective in blocking drug-induced CAV1 expression in HT29(US) cells, but not in DLD-1 cells. In HT29(US) cells, inhibition of the PI3K pathway using the Aurora kinase inhibitor A has been linked to the induction of autophagy [52] and this may explain why LY294002 blocked induction of CAV1 by both Methotrexate and Etoposide in HT29(US) but not DLD1 cells.

All the anti-neoplastic drugs employed, increased CAV1 expression in the colon cancer cell line HT29(US), although increases were most significant for Methotrexate and Etoposide. These were the only two drugs that increased significantly CAV1 protein levels in the other colon cancer cell line DLD-1 and the breast cancer cell line MCF7. Importantly, Methotrexate and Etoposide increased the mRNA and protein levels of CAV1 in colon and breast cancer cells. Several transcription factors are known to regulate the expression of CAV1 during cancer progression. In human lung cancer cells, CAV1 expression is up-regulated by PEA3/E1AF and down-regulated by Net/Elk-3 [53]. Also, activation of PPARγ correlates with an increase in CAV1 mRNA in breast and colon cancer cells [54], and RANKL up-regulates CAV1 during osteoclastogenesis [55]. The *CAV1* gene was identified as one of the possible target genes of NF-κB [56]. Therefore, CAV1 up-regulation by exposure to the chemotherapeutic agents Methotrexate or Etoposide (the drugs we characterized in greater detail here) could be due to activation of one or several of these transcription factors, and additional studies are required to define those involved.

Methotrexate and Etoposide increased mRNA and protein levels of CAV1 in colon and breast cancer cells through activation of the MEK/ERK pathway and the subsequent increase in ROS levels. In neuronal cells, Etoposide and Doxorubicin increased ROS via FOXO3 activation, which alone suffices to trigger bursts of ROS and then apoptosis [22]. In primary human lung cells, TIG-3, via the Ras/MEK pathway, induced the expression of superoxide-generating NADPH oxidases (Nox), such as Nox4, leading to an increase in intracellular ROS levels [57]. Also, in monocytic cells, stimulation of the Toll-like receptor 4 (TLR4) and 2 (TLR2) induces the IRAK-ERK pathway that connects to p67 phox-Nox2 for ROS generation, which regulate IL-1β transcription and processing [58]. Taken together, these observations suggest that Nox family members could be involved in the mechanisms that up-regulate CAV1 expression after exposure to anti-neoplastic drugs. This is an intriguing possibility that needs to be addressed in future studies.

Upon upregulation, CAV1 can be phosphorylated on tyrosine 14 (Y14), by Src-family kinases. Phosphorylation of CAV1 at this site has been linked to increased anchorage-independent growth and cell migration by a mechanism dependent on Grb7 [59]. Matrix metalloproteinase activation has been described in large cell lung carcinoma, where elevated CAV1 expression correlates with advanced stages of lung cancer as well as increased MMP9/MMP2 expression and activity. Knockdown of CAV-1 in H460 and 9D5 cells decreases the protein levels, as well as MMP9/MMP2 activity [60]. Moreover, cell invasion [61] and expression of the matrix metalloproteinase genes MMP1 and MMP2 are significantly enhanced by CAV1 in glioblastoma cells [62]. Here we show that pre-treatment with an inhibitor of the Src-family kinases (PP2) blocked the increase in cell migration and invasion in colon and breast cancer cells and that CAV1 silencing decreased particularly MMP9 activity induced by the anti-neoplastic drugs. Available evidence indicates that cytosolic ROS activate Src-family kinases [63], which may phosphorylate CAV1 [64] to enhance cell migration. Additional targets of cytosolic ROS are the Focal Adhesion Kinase (FAK) [65] and structural proteins such as β-actin [66]. Both Src and FAK are initiators of focal adhesion formation in adherent cells, which upon activation favor cell spreading and migration [67].

Further studies are required to define the mechanisms by which the increase in ROS production induced by Methotrexate or Etoposide promote cell migration in a CAV1-dependent manner. However, given that ROS activate Src-family kinases and the inhibitor PP2 reduces CAV1-enhanced migration, these kinases are likely to be involved. Moreover, we have previously shown that CAV1 phosphorylation on tyrosine-14 leads to activation of a novel Rab5-Tiam1-Rac1 signalng axis important in migration and invasion of cancer cells [31]. Consistent with the possibility that this pathway may be relevant here, transfection with the dominant negative Rac(S17N) mutant completely abolished Methotrexate- and Etoposide-induced DLD-1 invasion. Rather surprisingly, however, while the Tiam-1/Trio inhibitor (NSC23766) effectively reduced invasion of both HT29(US) and DLD-1 cells observed after Etoposide exposure, this was not the case following Methotrexate treatment, indicating that other Rac1 GEFs are likely to be involved downstream of CAV1.

Augmented CAV1 expression is frequently linked to enhanced metastatic potential of cancer cells (reviewed in [1, 7, 36]). As anticipated, knock-down of CAV1 efficiently reduced infiltation of several organs (liver, kidney, spleen) by HT29(US) cells in the carcinomatosis assay. However, the treatment with Methotrexate or Etoposide prior to injection of cells did not increase metastasis in this manner. Thus, we also evaluated accumulation of cells in the ascites fluid, which is a more sensitve marker and observed for Methotrexate treated cells a highly significant increase in cell number, while for Etoposide a trend was apparent but differences to controls were not significant (Figure 11B). It is interesting to speculate here that the pronounced difference observed for Methotrexate may relate to the increased relevance of PI3K activation in CAV1 induction in HT29(US) cells (Figure 5A).

Augmented CAV1 expression has been associated previously with progression towards a more aggressive cancer phenotype, for instance for prostate cancer and melanomas [38, 68–70], although in those cases the precise mechanisms involved were not defined. The novelty of the findings reported here resides in showing that exposure of cancer cells to anti-neoplastic drugs at sub-cytotoxic concentrations may induce signaling events and changes in transcription that favor a more aggressive metastatic phenotype in the absence of selection for drug-resistance. Whether similar mechanisms are relevant in cases where CAV1 upregulation occurs in the absence of drug exposure remains to be defined.

**Figure 12:**
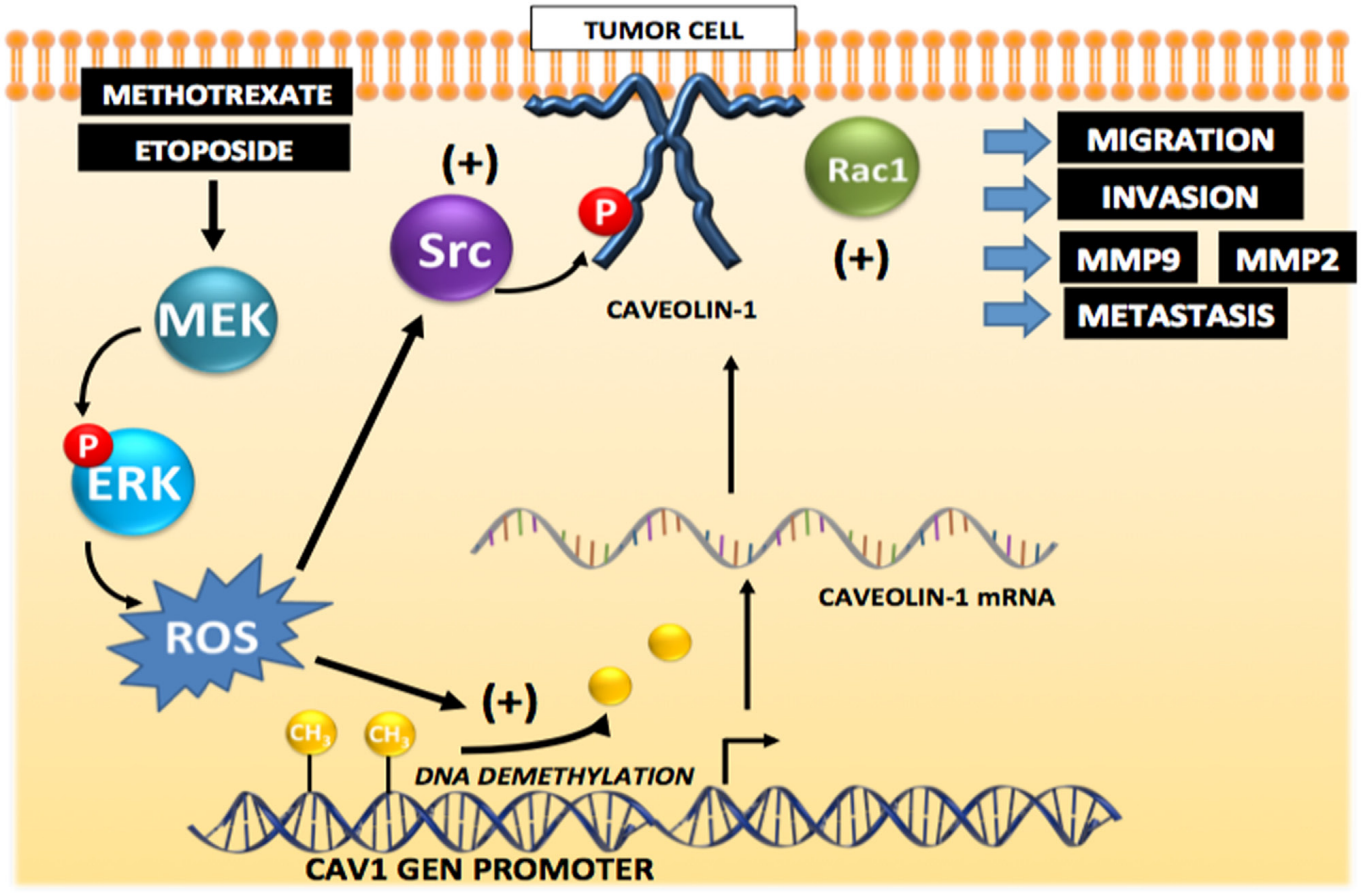
Working model summarizing the main findings described in this study identifying the mechanisms by which cytotoxic drugs induce CAV1 expression Initially, CAV1 expression is repressed by methylation in the gene promoter region in tumor cells. Exposure to the anti-neoplastic drugs Methotrexate or Etoposide induces promoter demethylation that increases transcription and expression of CAV1 that is is mediated by ERK activation and ROS production. Likewise, ROS are shown to promote Src family kinase-dependent CAV1 phosphorylation that may lead to Rac1 and metalloproteinase activation, as well as increased migration, invasion and metastasis.

We propose the working model shown in Figure 12, which is consistent with most of the data shown here. In breast and colon cancer cells, CAV1 expression is repressed by methylation; however, exposure to Methotrexate or Etoposide, and presumably other cytotoxic drugs, induces *CAV1* transcription, likely via promoter demethylation, and CAV1 expression mediated by ERK phosphorylation/activation and ROS production. Additionally, ROS are shown to promote Src-family kinase activation, CAV1 phosphorylation on Y14 and downstream Rac1 activation, which is indicated here as being responsible for metalloproteinase activation, increased migration, invasion and metastasis.

## MATERIALS AND METHODS

### Materials

Rabbit polyclonal anti-caveolin-1 (Transduction Laboratories, Lexington, KY, USA), anti-actin (R&D Systems, Minneapolis, MN, USA), goat polyclonal anti-MMP2 and anti-MMP9 (Santa Cruz Biotechnology, Santa Cruz, CA, USA), antibodies were used as indicated by the manufacturers. Goat anti-rabbit and goat anti-mouse IgG antibodies coupled to horseradish peroxidase (HRP) were from Merck-Millipore (Billerica, Massachusetts, USA) and KPL Laboratories (Washington DC, USA), respectively. The anti-neoplastic drugs Methotrexate, Etoposide, Doxorubicin, Staurosporine and Cisplatin were from Calbiochem (La Jolla, CA, USA), while Taxol was from Molecular Probes (Eugene, OR, USA). CpG methyltransferase was from New England BioLabs (Ipswich, MA, USA). The ECL chemiluminescent substrate and the BCA protein determination kit were from Pierce (Rockford, IL, USA). The Plasmid Midi Kit was from Qiagen (Valencia, CA, USA). The Quant-iT™ dsDNA Assay Kit was from Broad Range (Invitrogen (Carlsbad, CA, USA). Human fibronectin was from Becton Dickinson (San Jose, CA, USA). Hygromycin was from Calbiochem (La Jolla, CA, USA). Fetal bovine serum (FBS) was from Biological Industries (Cromwell, CT, USA). Cell culture media and antibiotics were from GIBCO (Invitrogen, Carlsbad, CA, USA). LY294002, PD98059 and Trolox were from Enzo Life Sciences (Farmingdale, NY, USA). The Histostain Bulk kit was from Zymed Laboratories (San Francisco, CA, USA) and the EZ DNA Methylation kit from Zymo Research (Orange, CA, USA).

### Cell culture

The colon cancer cell line HT29(US) is a metastatic derivative of HT29(ATCC) cells from ATCC (ATCC HTB-38) that we have employed previously [31, 71]. HT29(US) and HT29(ATCC) cells were cultured in high-glucose DMEM, DLD-1 colon cancer cells in RPMI and MCF7 breast cancer cells in DMEM-F12. All media were additionally supplemented with 10% FBS, 100 U/ml of penicillin and 100 μg/mL of streptomycin sulfate. Cells were cultured at 37°C in a humidified atmosphere containing 5% CO_2_.

### CAV1 shRNA lentiviral infection

Lentiviral transduction particles encoding for shRNA against CAV1 (sh-Cav-1 (#5)) and with a shRNA control (sh-Scramble) were obtained from the Broad Institute, Cambridge, USA and employed as previously described (Urra et al., 2012). Briefly, HEK293T cells were transfected with plasmids for the vector (pLKO.1puro), packaging (Δ8.9–pCMVΔR8.9 and vsv-g–pHCMV-G) and the corresponding shRNA against CAV1 (shCav-1(#5)) and luciferase (shLuc) using the Superfect^®^ Reagent (Qiagen^®^, Valencia, USA). Cell supernatants were recovered after 24 h, aliquoted and stored frozen at −20°C. The shRNA sequences tested were: GCTTCCTGATTGAGATTCAGT (shCav-1(#5)) and CGCTGAGTACTTCGAA ATGTC (shLuc). Cells (5 x 10^5^) were plated and transduced with lentivirus containing the indicated shRNAs and selected in puromycin-containing (1 μg/mL) cell culture medium for 1 week.

### Treatments with methylation and acetylation inhibitors

Cells (5 x 10^5^) were seeded in 60 mm plates 24 h before treatment with the methylation inhibitor, 5-aza dideoxycytidine (1, 2, 5, 10 μM) for 72 h or with the acetylation inhibitor, Trichostatin (50 ng/ml) for 24 h. Cells were also treated with a combination of 2.5 μM 5-aza dideoxycytidine (72 h) and 25 ng/mL Trichostatin for 24 h.

### Methylated DNA immunoprecipitation (MedIP)

MedIP was performed as has been described previously [72, 73] Briefly, genomic DNA (gDNA) was isolated from cell samples following overnight Proteinase K treatment in lysis buffer (20 mM Tris pH 8.0, 4 mM EDTA, 20 mM NaCl and 1% SDS) by phenol-chloroform extraction and subsequent ethanol precipitation in the presence of RNase. Purified gDNA was then sonicated to produce fragments of ∼400 bp. Fragmented gDNA (4 μg) was denatured for 10 min at 95°C and then immunoprecipitated overnight at 4°C with 4 μg of anti-5-methylcytidine (Eurogentec) antibody in a final volume of 500 μl of IP buffer (10 mM sodium phosphate pH 7.0, 140 mM NaCl, 0.05% Triton X-100). The mixture was incubated with 40 μl of dynabeads anti-mouse IgG (Invitrogen #11202D) for 2 h at 4°C and washed twice with 700 μl of IP buffer. Dynabeads were then treated with 7 μl of proteinase K (10 mg/ml) in 250 μl of Digestion buffer (50 mM Tris pH 8.0, 10 mM EDTA and 0.5% SDS) for 3 h at 50°C. Immunoprecipitated DNA was recovered by phenol-chloroform extraction followed by ethanol precipitation using glycogen as a carrier. Purified DNA was then evaluated using qPCR analysis, defining the enrichment levels as percentage of input material. Distinct primers were used to analyze the CAV1 proximal promoter (CAV1: Forward-GCCTTGGTTGCCCATACT; Reverse-CTAGGCACATCCCCAAGGT) or negative (CSa: Forward-ACATATCCAAGGACGTGTAA; Reverse-AGCTAACTCCAACTTTCCAG) and positive (IAP: Forward-TTGGGACAGTCCAAGTCTT; Reverse-CCCTTCACCACCTTCTTGAT) control regions.

### Western blotting

Cells were rinsed and harvested in ice-cold PBS containing 1 mM orthovanadate, 10 μg/ml benzamidine, 2 μg/ml antipain, 1 μg/ml leupeptin and 1 mM phenylmethyl-sulphonylfluoride (Ova-BAL-PMSF). Cells were then centrifuged at 3000 x g for 2 minutes at 4°C and the respective cell pellets were lysed by sonication in extraction buffer (Hepes 20 mM pH 7.4, NP40 0.1% and SDS 0.1% plus Ova-BAL-PMSF). Protein concentrations in extracts was determined using the BCA protein assay kit. Protein samples were separated by SDS-PAGE (50 μg per lane), transferred to nitrocellulose, blocked in PBS containing 5% non-fat milk and probed overnight at 4°C with anti-CAV1 antibody (1:5000) diluted in PBS containing 5% gelatin and 1% Tween-20. Protein loading in each lane was assessed by probing with an anti-β-actin antibody (1:5000). Goat anti-rabbit IgG antibodies coupled to horseradish peroxidase were used to detect bound first antibodies by EZ-ECL. Protein bands were quantified by densitometric analysis using the ImageJ 1.34s software (available from NIH at http://rsb.info.nih/ij/).

### Analysis of mRNA levels by quantitative real time qPCR

Total RNA was isolated with the reagent TriZOL^®^ reagent (Ambion, Life Technologies), following instructions provided by the manufacturer. Quality of RNA samples was corroborated by electrophoresis on 1% agarose gels and the concentration was determined using a Nanoquant infinite M200Pro instrument. RNA was treated with RNAase-free DNAase and employed as template for cDNA synthesis using M-MLV Reverse Transcriptase (Promega). PCR-amplification of CAV1 cDNA was performed combining the forward primer 5´-TGGTTTTACCGCTTGCTGTCTG with the reverse primer 5´-GCAAGTTGATGCGGACATTGCT. For amplification of β-actin cDNA used as a housekeeping control, the forward primer 5´-TGGCACCCAGCACAATGAAGA and reverse primer 5´-GAAGCATTTGCGGTGGACGAT-3´) were used. Primers were designed using Mx-Pro – Mx3000P v4.10 software.

Real-time PCR was performed using the Stratagene Mx3000p Real-Time PCR System and the Brilliant II SYBR Green Master Mix qPCR kit with 2 μl template cDNA in a final volume of 20 μl. The reaction cycle consisted of a first step for 10 min at 95°C followed by 40 cycles of consecutive 15-second steps at 95°C, 60°C and 72°C. Fluorescence emitted at 72°C was measured at the end of each cycle. After completion of all the amplification cycles, a melting curve analysis was run. DNA was quantified using the qPCR instrument Mx3000P (Agilent Technologies) and the results were analyzed with the MxPro v4.1 d software (Agilent Technologies).

### ROS determination by flow cytometry

Cells (3 x 10^4^) were seeded in 24-well plates 24 h before pre-treatment with 50 μM MEK inhibitor PD98059 (Enzo life science, BML-EI360-0005) for 30 min, followed by treatment with 100 nM Methotrexate for 20 h or 10 μM Etoposide for 0,16, 20, 22 or 24 h. Cells were washed 3 times with PBS and incubated with trypsin for 5 min. In suspension, the cells were loaded with 1.4 μg/ml DHR 123 (Invitrogen, D23806) in RPMI media without serum for 30 min at 37°C. The reaction was stopped on ice. Oxidation of DHR 123 to Rhodamine 123 was determined by flow cytometry (FACSCanto, Beckton Dickinson) at 515 nm.

### Migration and invasion assays

Cell migration was evaluated in Boyden Chamber assays (Transwell Costar, 6.5-mm diameter, 8-mm pore size), whereas invasion was evaluated in Matrigel assays (BD Biosciences, 354480), as reported previously [31].

### Metalloproteinase activity using zymography assays

Conditioned media from DLD1 and HT29(US) cells treated with 100 nM Methotrexate or 10 μM Etoposide for 48 h and afterwards serum-starved during 16 h were analyzed by zymography to determine the enzymatic activity of MMP-2 and MMP-9. For zymography experiments cell extracts were separated in 10% polyacrylamide gels copolymerized with gelatin (1 mg/ml). Samples (35 μg protein) were incubated for 30 min in sample buffer 5X (0.4M TrisHCl (pH 6.8) containing 5% sodium dodecyl sulfate (SDS), 20% glycerol, 0.03% bromophenol blue) under non-reducing conditions at room temperature. As an internal MW standard, we used 20 ng each of the pure latent and active forms of MMP-2 and MMP-9 (Chemicon, Temecula, CA). After electrophoresis, gels were incubated in 2.5% Triton X-100 for 30 minutes at room temperature and then for 24 h in metalloproteinase test buffer (150 mM Tris-HCl (pH 7.5), 150 mM NaCl, 5 mM CaCl2, 0.02% NaN3) at 37°C. Gels were fixed and stained in Coomassie blue R 250 for 3 h and rinsed overnight in distilled water. Gelatinase activity was identified as clear bands against a blue background [74].

### Animal studies

#### Bioethics statement

All animal experiments were conducted in accordance with the guidelines of CONICYT, Chile and approved by the Bioethics Committee of Fundación Ciencia & Vida. Balb C NoD SciD mice from the Jackson Laboratory were maintained under specific pathogen-free conditions and used at 6–8 weeks of age. To generate intraperitoneal carcinomatosis, 1 x 10^6^ HT29(US) sh-Scramble or sh-CAV1(#5) cells were treated with 100 nM Methotrexate (MT) or 10 μM Etoposide (ET) for 48 h. Then, cells in 100 μl saline solution were injected into the intraperitoneum of Balb C NoD SciD mice. Experiments involved 6 groups with 5 mice per group; (1) sh-Scramble basal, (2) sh-Scramble MT, (3) sh-Scramble ET, (4) sh-CAV1 (#5) basal, (5) sh-CAV1 (#5) MT, (6) sh-CAV1 (#5) ET. Between days 18-20 post-injection, the animals were evaluated according to the behavior punctuation scheme [43, 44]. After 21 days, the animals in groups (2) and (3) showed delays in their movements and appeared depressed with higher Morton Punctuation [43]. For these reasons, all mice in the different groups were euthanized at day 21 and evaluated for malignant paracentesis by counting the number of live cells in the ascites fluid. Solid tumor masses localized in spleen, pancreas, liver and kidney tissue, were removed and fixed in 10% formalin to visualize and determine total tumor mass.

### Statistical analysis

All data are expressed as mean ± standard error of mean (SEM) of three independent experiments. Data were analyzed using the unpaired t-test. Significance (*p*-value) was set at the nominal level of *p*<0.05 or less. All data were processed using INSTAT v. 3.05 (GraphPad Software, San Diego, USA, http://www.graphpad.com).

## SUPPLEMENTARY MATERIALS FIGURES AND TABLE



## Abbreviations

CAV1: caveolin-1
MDR: multidrug resistance
ROS: reactive oxygen species
Y14: tyrosine 14
