# Supplementary material for: Anti-neoplastic drugs increase caveolin-1-dependent migration, invasion and metastasis of cancer cells
Source: Oncotarget. 2017 Dec 5;8(67):111943–65. doi: 10.18632/oncotarget.22955 (PMC5762371; doi:10.18632/oncotarget.22955)
Supplement: Supplementary file 1 [file oncotarget-08-111943-s001.pdf]

Anti-neoplastic drugs increase caveolin-1-dependent migration, invasion and metastasis of cancer cells

SUPPLEMENTARY MATERIALS

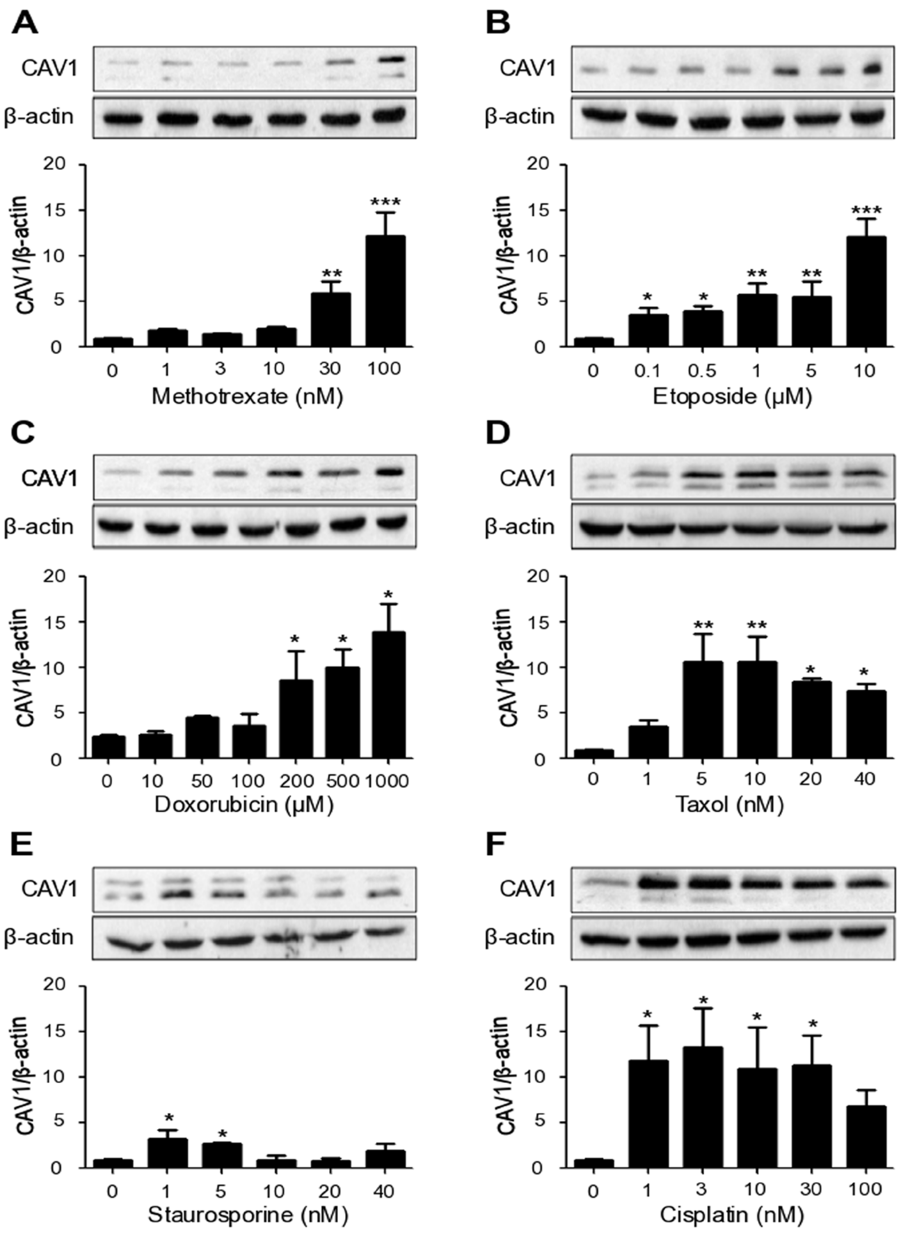

Supplementary Figure 1: Dose response curve for treatments with anti-neoplastic drugs. Colon cancer cells HT29(US) were treated with different concentrations of **(A)** Methotrexate, **(B)** Etoposide, **(C)** Doxorubicin, **(D)** Taxol, **(E)** Staurosporine for 48 h or **(F)** Cisplatin for 24 h. Cells were harvested and total protein extracts were separated by SDS-PAGE (50 μg total protein per lane) and analyzed by Western blotting with antibodies against CAV1 and β-actin. The graphs show the expression of CAV1 normalized to β-actin (mean ± SEM) of 3 independent experiments. Significant differences in comparison with the untreated condition (without drug) are indicated $^{***}p \leq 0.001$, $^{**}p \leq 0.01$, $^{*}p \leq 0.05$.

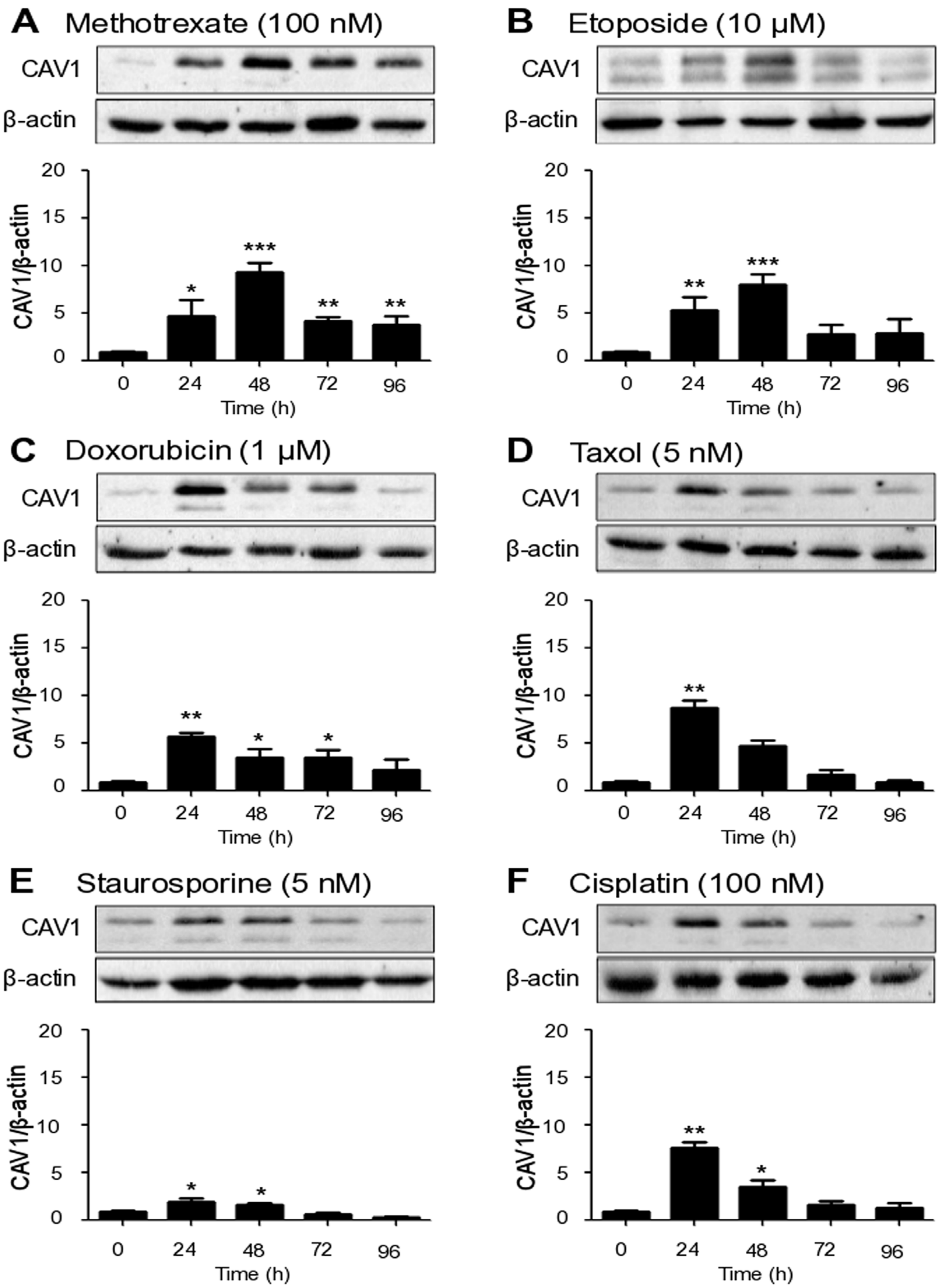

Supplementary Figure 2: Time course of treatment with anti-neoplastic drugs. HT29(US) colon cancer cells were treated for the indicated time periods with **(A)** 100 nM Methotrexate, **(B)** 10 μM Etoposide, **(C)** 1 μM Doxorubicin, **(D)** 5 μM Taxol, **(E)** 5 nM Staurosporine or **(F)** 100 nM Cisplatin. Cells were harvested and total protein extracts were separated by SDS-PAGE (50 μg total protein per lane) and analyzed by Western blotting with antibodies against CAV1 and β-actin. The graphs show the expression of CAV1 normalized to β-actin (mean ± SEM) of 3 independent experiments. Significant differences in comparison with the untreated condition (Time 0) are indicated $^{***}p \leq 0.001$, $^{**}p \leq 0.01$, $^{*}p \leq 0.05$.

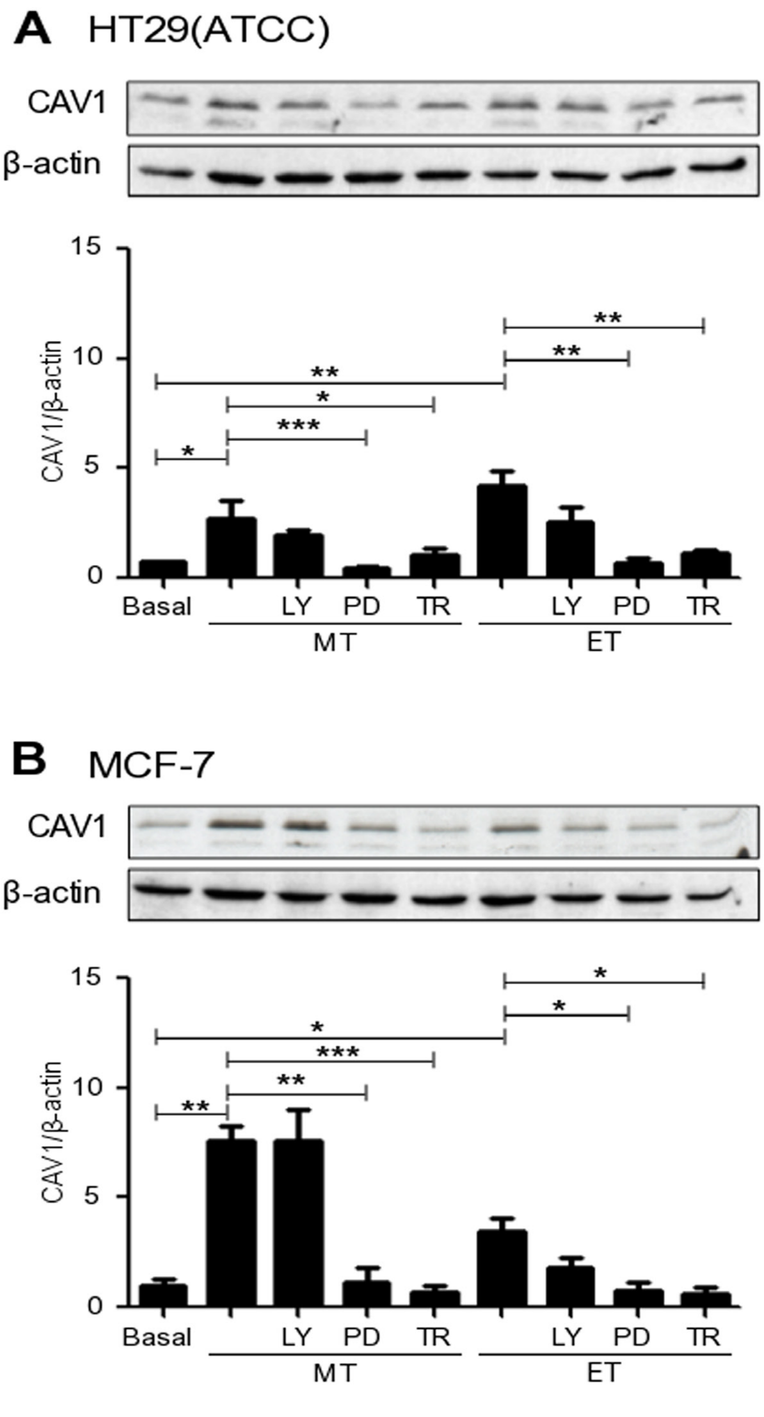

Supplementary Figure 3: Effect of MEK and PI3K inhibition or anti-oxidant treatment on CAV1 up-regulation induced by anti-neoplastic drugs in colon and breast cancer cells. **(A)** HT29(ATCC) colon and **(B)** MCF7 breast cancer cells were pre-treated with the PI3K inhibitor, LY394002 (LY, 10 µM), the MEK inhibitor, PD98059 (PD, 50 µM) or the vitamin E analog, Trolox (TR, 2 mM) for 30 min before treatment with 100 nM Methotrexate or 10 µM Etoposide for 48 h. Cells were harvested and total protein extracts were separated by SDS-PAGE (50 µg total protein per lane) and analyzed by Western blotting with antibodies against CAV1 and β-actin. The graphs show the expression of CAV1 normalized to β-actin (mean ± SEM) of 3 independent experiments. Significant differences in comparison with the untreated condition (Basal) are indicated $^{***}p \leq 0.001$, $^{**}p \leq 0.01$, $^{*}p \leq 0.05$.

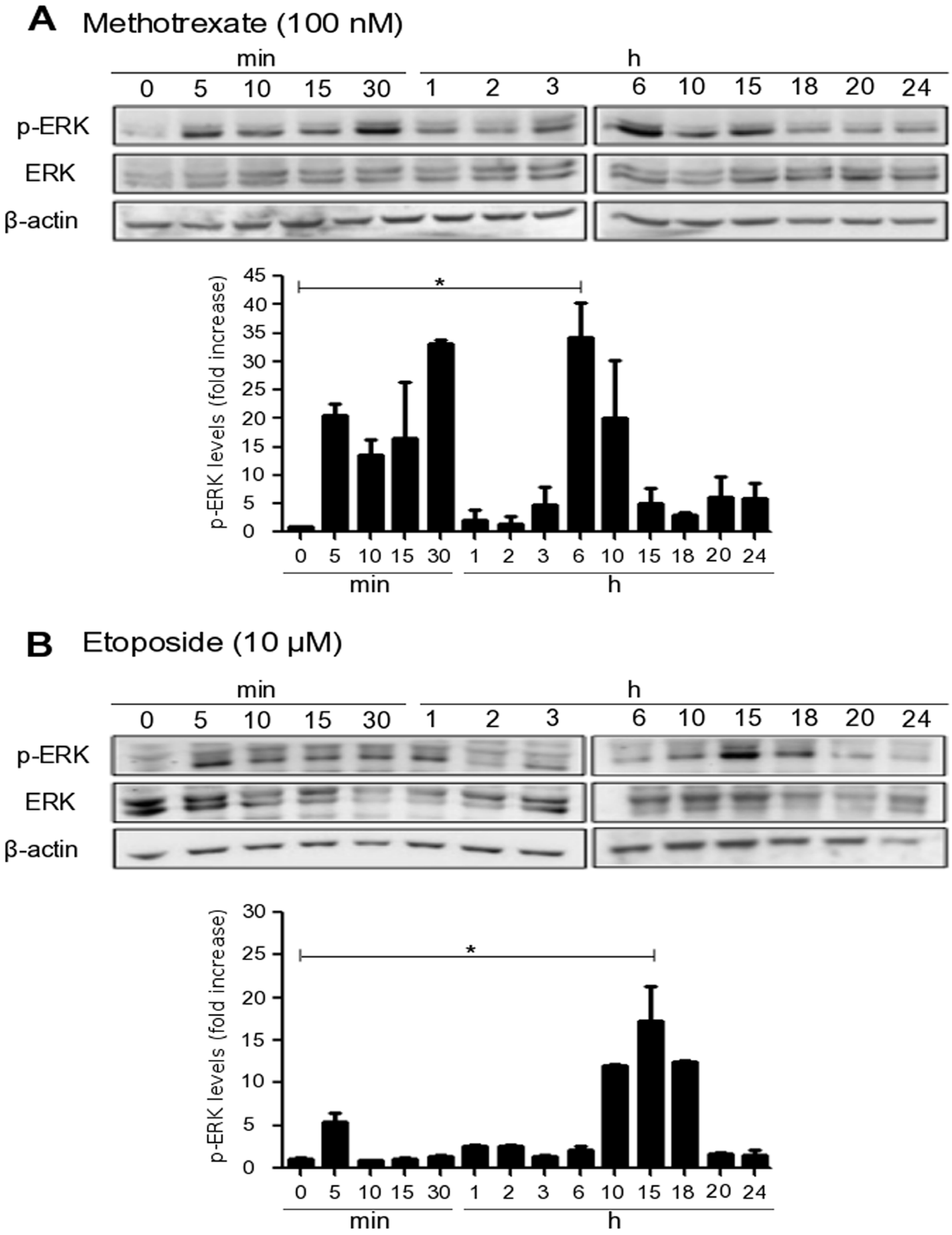

Supplementary Figure 4: ERK phosphorylation induced by anti-neoplastic drugs in colon cancer cells. DLD-1 cells (6 x 10^5) were seeded and 24 h later cells were treated for different periods of time with **(A)** 100 nM Methotrexate or **(B)** 10 µM Etoposide. Cells were harvested and total protein extracts were separated by SDS-PAGE (50 µg total protein per lane) and analyzed by Western blotting with antibodies against phosphorylated ERK, total ERK and β-actin. The graphs show the levels of phosphorylated ERK normalized to total ERK (mean ± SEM) of 3 independent experiments. Significant differences in comparison with the untreated condition (time 0) are indicated $^*p \leq 0.05$.

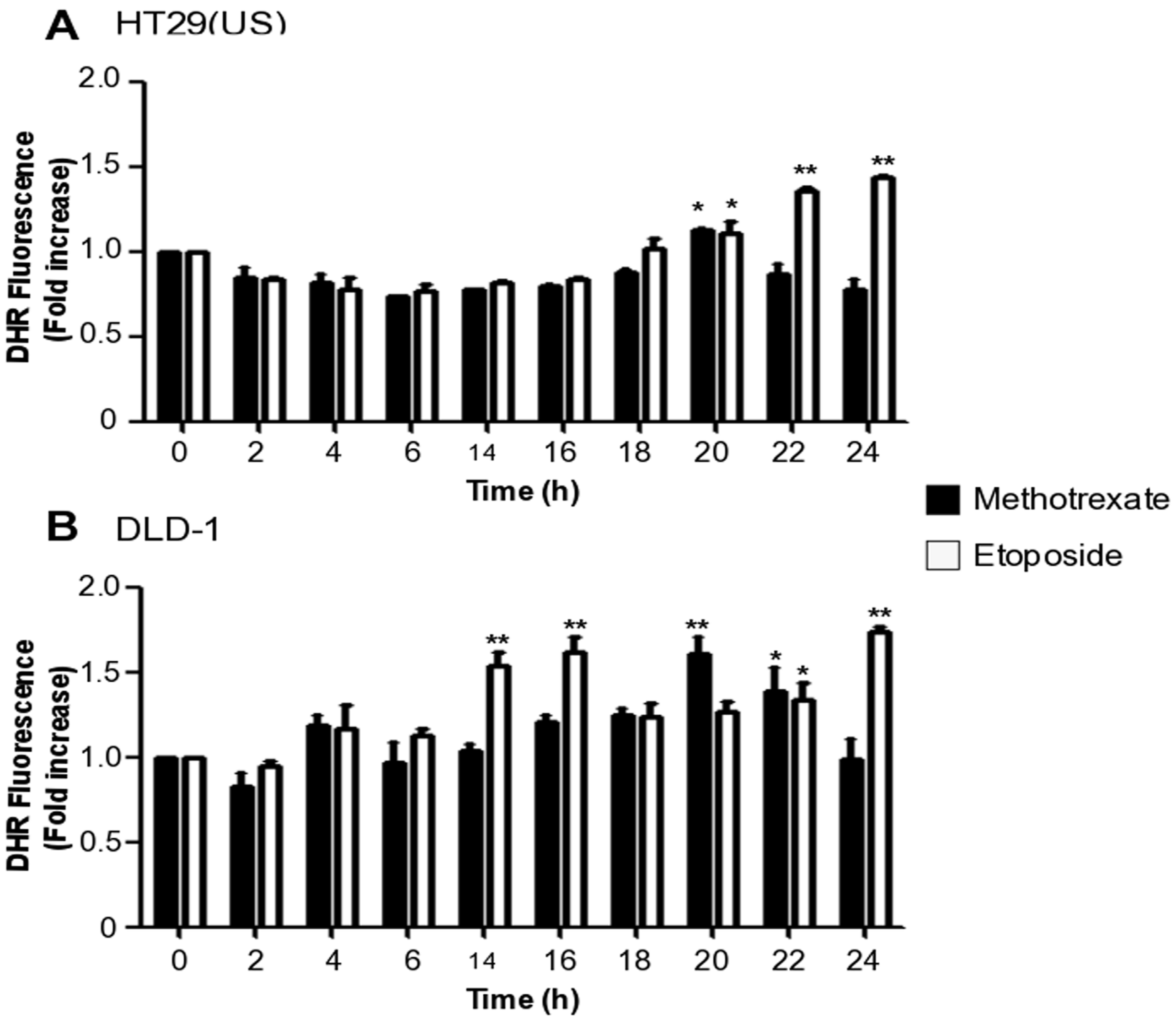

Supplementary Figure 5: Methotrexate and Etoposide induce ROS production in colon cancer cell lines. (A) HT29(US) or **(B)** DLD-1 cells (3 x 10^4) were seeded in 24-well plates 24 h before treatment for different periods of time with (A) 100 nM Methotrexate or (B) 10 µM Etoposide. Cells were loaded with the probe DHR 123 (1.4 µg/ml) for 30 min at 37°C. The reaction was stopped by placing the cells on ice. Oxidation of DHR 123 to Rhodamine 123 was determined by flow cytometry at 515 nm. The graphs show ROS levels (mean ± SEM) averaged from 3 independent experiments. Significant differences in comparison with the untreated condition (time 0) are indicated $^{**}p \leq 0.001$, $^{*}p \leq 0.05$.

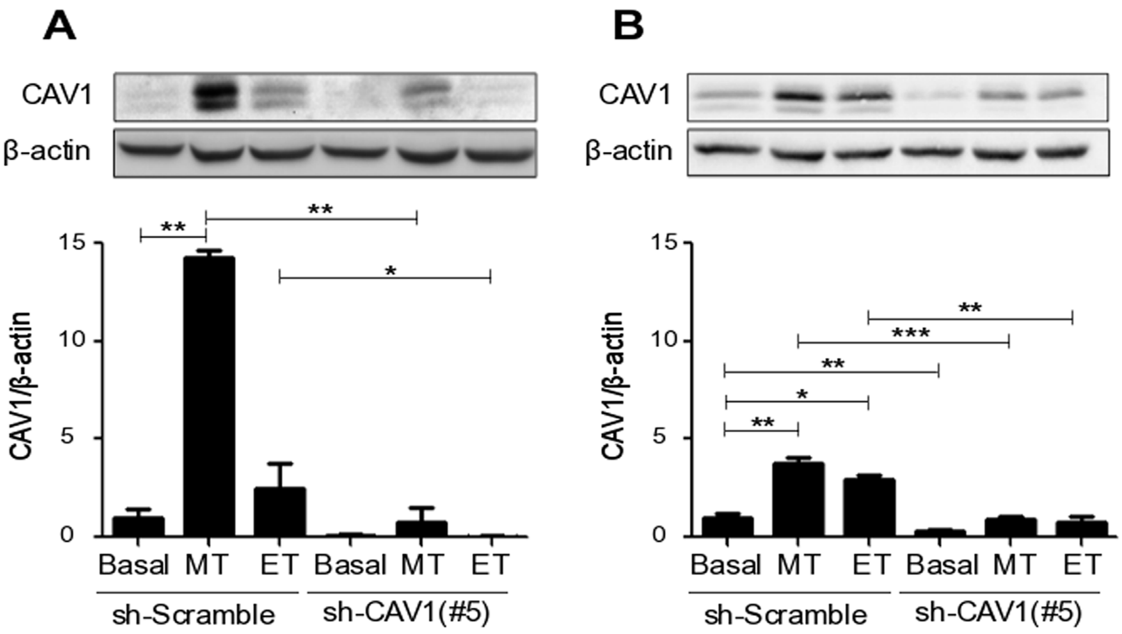

Supplementary Figure 6: CAV1 silencing by shRNA in colon cancer cell lines. (A) HT29(US) or **(B)** DLD-1 were transduced with lentiviral particles encoding shRNA against CAV1 (sh-Cav-1 (#5)) or control shRNA (sh-Scramble). Cells were harvested and total protein extracts were separated by SDS-PAGE (50 µg total protein per lane) and analyzed by Western blotting with antibodies against CAV1 and β-actin. The graphs show CAV1 expression normalized to β-actin (mean ± SEM) of 3 independent experiments. Significant differences in comparison with the untreated condition (Basal) are indicated $^{***}p \leq 0.001$, $^{**}p \leq 0.01$, $^{*}p \leq 0.05$.

Supplementary Table 1: Cell viability after the treatment with anti-neoplastic drugs

A. Percentage of viable cells

Cell line	Antineoplastic drugs						
	NT	MT	ET	DX	TX	ST	CP
HT29(US)	83 ± 3.7	67 ± 1.5	62 ± 6.2	5 ± 0.3	80 ± 1.9	81 ± 0.3	88 ± 0.3
HT29(ATCC)	91 ± 2.7	89 ± 1.1	80 ± 0.3	4 ± 0.1	95 ± 0.5	93 ± 0.6	94 ± 0.4
DLD1	90 ± 1.3	70 ± 3.0	46 ± 2.2	9 ± 0.4	77 ± 3.1	75 ± 2.1	79 ± 2.0
MCF7	64 ± 3.1	63 ± 4.3	87 ± 0.1	3 ± 0.8	60 ± 2.5	68 ± 3.2	79 ± 1.2

B. Percentage of non-viable cells

Cell line	Antineoplastic drugs													
	NT		MT		ET		DX		TX		ST		CP	
	A	N	A	N	A	N	A	N	A	N	A	N	A	N
HT29(US)	9 ± 0.4	8 ± 3.3	18 ± 0.7	15 ± 1.0	23 ± 6.7	15 ± 3.6	84 ± 0.5	11 ± 0.9	14 ± 2.2	6 ± 0.3	12 ± 1.0	7 ± 0.7	8 ± 0.8	5 ± 1.0
HT29(ATCC)	6 ± 2.7	3 ± 0.1	7 ± 0.41	4 ± 0.7	14 ± 0.1	7 ± 0.3	91 ± 0.6	5 ± 0.5	2 ± 0.2	3 ± 0.3	3 ± 0.2	4 ± 0.4	3 ± 0.3	3 ± 0.7
DLD1	9 ± 1.4	1 ± 0.1	25 ± 5.9	6 ± 2.9	46 ± 2.3	7 ± 0.1	87 ± 0.9	4 ± 0.5	21 ± 3.2	2 ± 0.0	24 ± 1.9	2 ± 0.1	20 ± 1.8	1 ± 0.3
MCF7	16 ± 0.1	20 ± 3.1	16 ± 0.2	21 ± 4.2	9 ± 0.2	4 ± 0.3	89 ± 1.0	8 ± 0.3	14 ± 0.3	26 ± 2.1	13 ± 0.9	9 ± 2.3	11 ± 0.4	10 ± 1.6

Colon cancer cells HT29(US), HT29(ATCC) and DLD-1 and breast cancer cells MCF7 were treated with 100 nM Methotrexate (MT), 10 µM Etoposide (ET), 1 µM Doxorubicin (DX), 5 nM Staurosporine (ST), 5 nM Taxol or 100 nM Cisplatin (CP) for 48 h. Cells were stained with propidium iodide and cell viability was assessed by flow cytometry as previously described [42]. Section A shows the percentage of viable cells after each treatment and section B shows the percentage of non-viable cells apoptotic cells (A) and necrotic cells (N).
